# Association between loneliness and cognitive function, and brain volume in community-dwelling elderly

**DOI:** 10.3389/fnagi.2024.1389476

**Published:** 2024-04-29

**Authors:** Hunju Lee, Sang Yeol Yong, Hyowon Choi, Ga Young Yoon, Sangbaek Koh

**Affiliations:** ^1^Department of Preventive Medicine, Wonju College of Medicine, Yonsei University, Wonju, Republic of Korea; ^2^Institute of Genomic Cohort, Wonju College of Medicine, Yonsei University, Wonju, Republic of Korea; ^3^Department of Rehabilitation Medicine, Wonju College of Medicine, Yonsei University, Wonju, Republic of Korea; ^4^International Olympic Committee Research Centre Korea, Yonsei Institute of Sports Science and Exercise Medicine, Wonju, Republic of Korea; ^5^Department of Radiology, Wonju Severance Christian Hospital, Wonju College of Medicine, Yonsei University, Wonju, Republic of Korea

**Keywords:** neuroimaing, dementia, loneliness, cognition, neuropsychological test

## Abstract

**Introduction:**

We investigated the relationship between loneliness, cognitive impairment, and regional brain volume among elderly individuals residing in the Korean community.

**Methods:**

Data from the ARIRANG aging-cognition sub-cohort, collected between 2020 and 2022, were utilized for the present study. Loneliness was assessed using the UCLA-Loneliness Scale (UCLA-LS) questionnaire and the relevant item from Center for Epidemiologic Studies Depression Scale Korean version (CES-D-K). Cognitive impairment was measured through Mini-Mental State Examination (K-MMSE-2) and Seoul Neuropsychological Screening Battery (SNSB-C), with five sub-categories: attention, memory, visuospatial function, language, and executive function. Logistic regression was employed for prevalence ratios related to cognitive impairment, while linear regression was used for regional brain volume including white matter hyperintensity (WMH) and cortical thickness.

**Results:**

Our analysis involved 785 participants (292 men and 493 women). We observed increased cognitive impairment assessed by K-MMSE-2 [UCLA-LS: odds ratio (OR) 3.133, 95% confidence interval (CI) 1.536–6.393; loneliness from CES-D: OR 2.823, 95% CI 1.426–5.590] and SNSB-C total score (UCLA-LS: OR 2.145, 95% CI 1.304–3.529) in the lonely group compared to the non-lonely group. Specifically, the lonely group identified by UCLA-LS showed an association with declined visuospatial (OR 1.591, 95% CI 1.029–2.460) and executive function (OR 1.971, 95% CI 1.036–3.750). The lonely group identified by CES-D-K was associated with impaired memory (OR 1.577, 95% CI 1.009–2.466) and executive function (OR 1.863, 95% CI 1.036–3.350). In the regional brain volume analysis, loneliness was linked to reduced brain volume in frontal white matter (left: −1.24, 95% CI −2.37 ∼−0.12; right: −1.16, 95% CI −2.31 ∼ −0.00), putamen (left: −0.07, 95% CI −0.12 ∼−0.02; right: −0.06, 95% CI −0.11 ∼−0.01), and globus pallidus (−15.53, 95% CI −30.13 ∼−0.93). There was no observed association in WMH and cortical thickness.

**Conclusion:**

Loneliness is associated with cognitive decline and volumetric reduction in the frontal white matter, putamen, and globus pallidus.

## 1 Introduction

Over the past few centuries, global life expectancy has increased, leading to an aging population worldwide. Consequently, the prevalence of elderly related conditions associated with cognitive decline, particularly dementia and mild cognitive impairment, has significantly risen, posing social issues (Prince et al., 2016). Unlike other elderly related conditions, there is a lack of proven treatments for cognitive decline, making prevention before the onset of the disease crucial (Livingston et al., 2020).

Cognitive impairment is influenced by various factors such as smoking, physical inactivity, and social connection. Social connection is an umbrella term encompassing the structure, function, and quality of social relationships, including concepts like social network, social isolation, and loneliness (Holt-Lunstad, 2022). Loneliness, in particular, is a measurement of the functional component of social connection (Holt-Lunstad, 2018), defined as “a subjective and unwelcome feeling of lack or loss of companionship”(Perlman and Peplau, 1981). Loneliness has been associated with increased mortality (Holt-Lunstad et al., 2015) and various diseases such as cardiovascular disease, stroke, anxiety, and depression (Valtorta et al., 2016; Beutel et al., 2017). A meta-analysis conducted in 2019 found that loneliness increases the risk of dementia and moderate cognitive impairment (Lobo et al., 2008; Lara et al., 2019).

In addition to its association with various adverse health outcomes, the high prevalence of loneliness makes it even more important from a public health perspective. A study by Perissinotto et al. (2012), analyzing the Health and Retirement Study, found that 43% of older adults reported experiencing loneliness, while a survey conducted by the Kaiser Family Foundation and The Economist (DiJulio et al., 2018) indicated that 22% of American adults responded as feeling lonely. A meta-analysis investigating the prevalence of loneliness across 113 countries prior to the COVID-19 pandemic revealed that problematic levels of loneliness in older adults ranged from approximately 5.2%–21.3%, depending on the region (Surkalim et al., 2022). Former US surgeon general Vivek Murthy even coined the term “Loneliness epidemic” to describe this phenomenon (Murthy, 2017). In addition to these alarming statistics, there have been significant efforts to address loneliness. Both the UK (Department for Digital, Culture, Media and Sport, 2018) and Japan (Tomohiro Osaki, 2021) established ministries dedicated to addressing loneliness in 2018 and 2021, respectively, and various campaigns have been conducted worldwide (Campaign to End Loneliness, 2024; Coalition to End Social Isolation Loneliness, 2024; Ending loneliness together, 2024). Furthermore, the World Health Organization emphasized the importance of improving research and understanding the mechanisms underlying the health impacts of loneliness in its advocacy brief published in 2021 (Committee on the Health Medical Dimensions of Social Isolation Loneliness in Older Adults et al., 2020; World Health Organization, 2021).

Based on this interest, the public health community has made significant progress in understanding the biological mechanisms of loneliness, with one such attempt being to explain the impact of loneliness on cognitive function through changes in brain volume. For instance, Salinas et al. (2022) demonstrated that the group experiencing loneliness exhibited poorer executive function, lower total brain volume, and greater white matter injury. Similarly, Van Der Velpen et al. (2022) found that the baseline white matter volume was smaller in the lonely group. However, these studies have yielded inconsistent results, and they have the limitation of measuring loneliness using only a single item derived from the Center for Epidemiologic Studies Depression Scale (CES-D). Loneliness can be measured using both scales (e.g., UCLA-Loneliness Scale) and single items, with single items being more commonly used in large population samples. However, due to differences such as the directness of survey items, the UK Office for National Statistics recommends using both measurement methods (Snape and Martin, 2018). Additionally, most existing studies have been conducted in Europe and America, but considering that the experience of loneliness is influenced by cultural factors (Lykes and Kemmelmeier, 2014), there is a need to conduct research on a more diverse range of races and countries.

Therefore, the current study aims to investigate the impact of loneliness on cognitive impairment and brain volume in middle-aged and older Asian adults, using both UCLA-Loneliness Scale (UCLA-LS) and the single item from CES-D to assess loneliness.

## 2 Materials and methods

### 2.1 Participants

The present study utilized data from the Aging-cognition sub-cohort on ARIRANG cohort. The ARIRANG cohort is part of the Korean Genome and Epidemiology Study (KoGES) (Kim et al., 2017), conducted by the Korea Disease Control and Prevention Agency. It focuses on a cohort of residents in Wonju and Pyeongchang. The cohort initially established in 2005 with the aim of investigating the causes of cardiovascular diseases. However, as the participants were aging, the primary outcomes were changed to elderly diseases and cognitive function in 2020. Subsequently, from the existing cohort, 930 individuals aged 55–79 were randomly selected. Individuals with severe cognitive impairment who could not participate in the survey and medical test were excluded during the selection process. The cohort’s survey items include surveys (e.g., general information, medical history, social health, and others), anthropometric measurements (e.g., height, weight, and others), laboratory tests (e.g., clinical chemistry, complete blood count, and others), brain magnetic resonance imaging (MRI), and neuropsychological test (e.g., K-MMSE-2 and SNSB-C). Data was collected by trained investigators following a pre-established protocol, with surveys conducted every 3 years.

**FIGURE 1:**
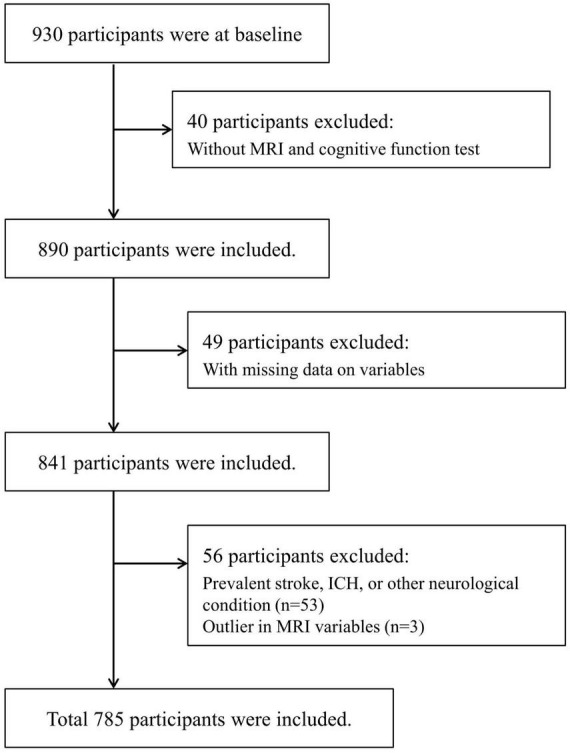
Flow chart of the eligible participants selection. ICH, intracranial hemorrhage; MRI, magnetic resonance imaging.

Recruitment for the Aging-cognition cohort took place from 2020 to 2022, with a total of 930 participants recruited. After excluding 145 individuals for various reasons, such as not undergoing MRI or neuropsychological testing (40 participants), having pre-existing neurological issues like stroke or hemorrhage (53 participants), unanswered survey questions (49 participants), and outliers in MRI data (Figure 1), the analysis was conducted on a final sample of 785 participants.

### 2.2 Loneliness

In the current study, loneliness was measured using the UCLA-LS and a specific loneliness-related item from the Center for Epidemiological Studies Depression Scale for Korean version (CES-D-K). The UCLA-LS is a survey designed to assess the degree of loneliness, comprising a total of 20 items (Russell, 1996). Each item is scored on a scale of 1 to 4, resulting in a total score range of 20–80. Higher scores indicate a higher level of loneliness. For the current study, individuals with UCLA-LS scores of 44 or above were classified into the group experiencing loneliness (Cacioppo and Patrick, 2008). The CES-D-K, originally developed to screen depression, includes items related to loneliness. Loneliness measurement based on the CES-D-K (CES-D-L) involved categorizing individuals who answered to the 14th item (“I felt lonely”) for 3 or more days per week as the group experiencing loneliness, following similar approaches in previous studies (Salinas et al., 2022).

### 2.3 Cognitive impairment

Cognitive function, assessed using the Korean version Mini-Mental State Examination 2 (K-MMSE-2) and Seoul Neuropsychological Screening Battery for Cognition Core (SNSB-C), was dichotomized into cognitively impaired and cognitively normal categories. The K-MMSE-2 is a tool commonly used for dementia screening, and a score below 24 is indicative of cognitive impairment. In addition, we conducted sensitive analyses with the cut off based on education level and age for K-MMSE-2. The SNSB-C is a comprehensive cognitive assessment tool covering attention, language, visuospatial function, memory, and executive function (Jahng et al., 2015). Specific tests within the SNSB-C included the Digit Span Test (forward + backward) for attention, Short form of the Korean-Boston Naming Test (S-K-BNT) for language ability, Rey Complex Figure Test (RCFT) for visuospatial ability, Seoul Verbal Learning Test for Elderly (SVLT-E) direct recall score for memory, and Korean version of Trail Making Test for Elderly (K-TMT-E): part B for executive function. The overall score of SNSB-C was calculated based on standardized criteria. For the memory section, the SVLT-E total score was used, and for the executive function section, scores from Digit Symbol Coding (DSC), Controlled Oral Word Association Test (COWAT): animal_giut, and Korean-Color Word Stroop Test 60 (K-CWST-60) were added. All SNSB-C scores were transformed into *T*-scores based on age and education level, with cognitive impairment considered if the score fell below 1 SD.

### 2.4 MRI metrics

Brain MRI scans were obtained using the Siemens Magnetom Skyra (3.0T; Siemens Healthineers, Erlangen, Germany), and the pre-established protocol was consistently followed throughout the current study period. The scan protocol included T1-weighted, T2-weighted, diffusion tensor imaging (DTI), functional magnetic resonance imaging (fMRI), and fluid-attenuated inversion recovery (FLAIR) sequences, with parameters developed by referencing the ADNI (Alzheimer’s Disease Neuroimaging Initiative) (Weiner et al., 2015). Sequences were validated by capturing sample images with volunteers or phantoms to ensure parameter integrity. The complete imaging protocol and parameters can be found in Supplementary Data 1 and Supplementary Table 1.

Prior to analysis, all scans underwent visual inspection for artifacts, and rescans were performed if artifacts were detected. Additionally, the Structural Similarity Index Map (SSIM) was used to assess image quality. Brain metrics were calculated using the CIVET (Lepage et al., 2021) and Fastsurfer (Henschel et al., 2020) deep learning models. Brain volume measurements included intracranial brain volume, total cerebral white/gray matter, white/gray matter for each lobe, basal ganglia, and limbic system volumes. Brain volume and cortical thickness were measured on T1-weighted image and white matter hyperintensity (WMH) volume was measured using FLAIR images. Total brain volume was assessed by the sum of cortical gray matter, subcortical gray matter, and cortical white matter.

Brain MRI reading was conducted by two radiologists. They evaluated the presence of lacune, microhemorrhage, large vessel stroke, intracerebral hemorrhage, tumor, hematoma, and hydrocephalus in each brain region. Brain MRI images underwent anonymization by removing names and patient numbers before analysis.

### 2.5 Covariates

Age was treated as a continuous variable, while gender was defined as a binary variable (men/women) based on respondents’ answers. The educational level was surveyed across nine categories, ranging from no formal education to graduate school. For analysis purposes, it was later re-categorized into three groups: below high school graduation, high school graduation, and college graduation or higher. Coexisting conditions included the presence or absence of hypertension, diabetes, and cardiovascular diseases. Hypertension was defined as having received a diagnosis from a doctor, or having a measured systolic blood pressure of 140 mmHg or higher, or a measured diastolic blood pressure of 90 mmHg or higher. Diabetes was defined as having received a diagnosis from a doctor, or having a measured fasting blood glucose level of 126 mg/dl or higher, or a measured HbA1c level of 6.5% or higher. Cardiovascular diseases were defined as having received a diagnosis of cardiovascular diseases from a doctor. Depression was defined as individuals scoring 16 or higher on the CES-D-K questionnaire.

### 2.6 Statistical analysis

Table 1 presents the basic characteristics of the study participants, with continuous variables presented as mean and SD, and categorical variables as counts and proportions. Age and MRI metrics were treated as continuous, while gender, education level, comorbidities, loneliness, and cognitive impairment were treated as categorical. Some brain volumetric variables were log-transformed to correct for skewness. Additionally, baseline characteristics of the loneliness group measured by UCLA-LS and CES-D-L were also provided (Supplementary Table 2 and Supplementary Figure 1). To investigate the association between loneliness and cognitive impairment, Chi-square tests were conducted (Supplementary Table 3 and Supplementary Figures 2, 3). Logistic regression analysis was performed to control for confounding variables, including age, gender, education level, and coexisting diseases (hypertension, diabetes, and cardiovascular diseases) (Table 2 and Supplementary Table 4). To examine the relationship between loneliness and structural brain changes, *T*-tests and linear regression analyses were conducted (Supplementary Tables 5, 6). In multiple linear regression analysis, adjustments were made for age, gender, education level, coexisting diseases (hypertension, diabetes, and cardiovascular diseases), and intracranial brain volume. A sensitivity analysis was also conducted to assess the impact of depressive symptoms (Supplementary Table 7). All statistical tests were two-sided, and significance was considered when the *p*-value was less than 0.05. Statistical analyses were performed using R version 4.2.2.

**TABLE 1:** Baseline characteristics for participants (*n* = 785).

Variables	Total (*n* = 785)	Older than 65 (*n* = 513)	Less than 65 (*n* = 272)
Age, mean ± SD	67.3 ± 6.3	71.0 ± 4.1	60.4 ± 2.7
**Gender, *n* (%)**			
Men	292 (37.2)	209 (40.7)	83 (30.5)
Women	493 (62.8)	304 (59.3)	189 (69.5)
**Education, *n* (%)**			
Under high school	358 (45.6)	275 (53.6)	83 (30.5)
High school	229 (29.2)	123 (24.0)	106 (39.0)
Upper college	198 (25.1)	115 (22.4)	83 (30.5)
**Comorbidities, *n* (%)**			
Hypertension	429 (54.6)	312 (60.8)	117 (43.0)
Diabetes	179 (22.8)	135 (26.3)	44 (16.2)
Cardiovascular disease	93 (11.8)	77 (15.0)	16 (5.9)
Depressive symptom	163 (20.8)	122 (23.8)	51 (15.1)
**Lonely group vs. not lonely group** ^1^			
Based on UCLA-LS, *n* (%)	132 (16.8)	100 (19.5)	32 (11.8)
Based on CES-D-L, *n* (%)	205 (26.1)	148 (28.8)	57 (21.0)
Cognitive impairment (based on K-MMSE-2), *n* (%)	35 (4.5)	30 (5.8)	5 (1.8)
**Cognitive impairment (based on SNSB-C), *n* (%)** ^2^			
Attention	164 (20.9)	97 (18.9)	67 (24.6)
Language	60 (7.6)	32 (6.2)	28 (10.3)
Visuospatial function	169 (21.5)	108 (21.1)	61 (22.4)
Memory	109 (13.9)	76 (14.8)	33 (12.1)
Frontal/executive function	53 (6.8)	38 (7.4)	15 (5.5)
Total	93 (11.8)	61 (11.9)	32 (11.8)
Total cerebral volume, mean ± SD	979.9 ± 89.16	965.85 ± 85.14	1,006.40 ± 90.69
Cerebral white matter volume, mean ± SD	156.41 ± 51.97	449.03 ± 50.12	470.33 ± 52.62
Cerebral gray matter volume, mean ± SD	193.62 ± 43.15	487.54 ± 41.91	505.08 ± 43.20
Subcortical gray matter volume, mean ± SD	29.88 ± 4.15	29.29 ± 4.29	30.98 ± 3.62
WMH volume, mean ± SD	3.43 ± 0.39	3.53 ± 0.40	3.25 ± 0.28

CES-D-L, loneliness scale from Center for Epidemiologic Studies Depression Scale; K-MMSE-2, Korean version of Mini-Mental State Examination 2; MRI, magnetic resonance imaging; SD, standard deviation; SNSB-C, Seoul Neuropsychological Screening Battery; UCLA-LS, UCLA-Loneliness Scale.

^1^Depressive symptom was measured by CES-D points over 16 points.

^2^SNSB-C group was by <1 SD.

**TABLE 2:** Logistic regression between loneliness and neuropsychological test.

Variables	K-MMSE-2				SNSB-C: total			
	Univariate		Multivariate		Univariate		Multivariate	
	OR (95% CI)	*p*-Value	OR (95% CI)	*p*-Value	OR (95% CI)	*p*-Value	OR (95% CI)	*p*-Value
UCLA-LS	3.133 (1.536, 6.393)	0.002	1.939 (0.901, 4.174)	0.090	2.145 (1.304, 3.529)	0.003	1.935 (1.161. 3.223)	0.011
CES-D-L	2.823 (1.426, 5.590)	0.003	2.081 (1.012, 4.276)	0.046	1.253 (0.780, 2.015)	0.351	1.106 (0.680, 1.799)	0.685

CES-D-L, loneliness scale from Center for Epidemiologic Studies Depression Scale; K-MMSE-2, Korean version of Mini-Mental State Examination 2; MRI, magnetic resonance imaging; SNSB-C, Seoul Neuropsychological Screening Battery; UCLA-LS, UCLA-Loneliness Scale. Age, gender, education level, hypertension, diabetes, and CVD were adjusted in multivariate analyses.

## 3 Results

### 3.1 Baseline characteristics

From 2020 to 2022, a total of 930 participants were enrolled in the study, of which 785 individuals were included in the analysis (Figure 1). The average age of the participants was 67.3 years, with 292 men (37.2%) and 493 women (62.8%). The prevalence of coexisting conditions was as follows: hypertension 54.6%, diabetes 22.8%, cardiovascular diseases 11.8%, and depression 20.8%. According to the UCLA-LS criteria, 16.8% of participants reported feeling lonely, while 26.1% reported loneliness based on the CES-D-L (Table 1).

Regarding cognitive impairment, 4.5% of participants scored below 24 on the K-MMSE-2, and 11.8% scored below 1 SD on the SNSB-C. Detailed SNSB-C subcategories revealed that 20.9% had decreased attention, 7.6% had impaired language ability, 21.5% had reduced visuospatial ability, 13.9% had memory decline, and 6.8% had executive function impairment—all falling below 1 SD for age and education level. The mean total cerebral volume was 979.9 cm^3^, the mean WMH volume was 3.43 mm^3^.

### 3.2 Association between loneliness and cognitive impairment

In the chi-square analysis examining the relationship between loneliness and cognitive impairment, the K-MMSE-2 showed a statistically significant association with loneliness assessed by both UCLA-LS and CES-D-L. The UCLA-LS demonstrated statistical significance not only with the K-MMSE-2 but also with the overall score of the SNSB-C. When breaking down cognitive impairment into specific subcategories, executive function showed significant associations with loneliness assessed by both UCLA-LS (*p*-value: 0.03) and CES-D-L (*p*-value: 0.03) (Supplementary Table 3).

In logistic regression analysis exploring the relationship between loneliness and cognitive impairment, univariate analysis revealed that loneliness assessed by both UCLA-LS and CES-D-L increased the odds of scoring below 24 on the K-MMSE-2, with statistical significance [UCLA-LS odds ratio (OR) 3.133, confidence interval (CI) 1.536–6.393; loneliness from CES-D OR 2.823, CI 1.426–5.590]. The relationship with the total score of SNSB-C was statistically significant only with UCLA-LS (OR 2.145, CI 1.304–3.529). These associations remained statistically significant for all relationships, except for the relationship between UCLA-LS and K-MMSE-2, even after adjusting for gender, age, education level, and coexisting diseases (Table 2).

In specific cognitive domains, loneliness measured by UCLA-LS was associated with a decreased visuospatial ability (OR 1.591, 95% CI 1.029–2.460), while loneliness assessed by CES-D was associated with a decline in memory (OR 1.577, 95% CI 1.009–2.466). Both UCLA-LS and CES-D-L were associated with a decreased executive function (UCLA-LS: OR 1.971, 95% CI 1.036–3.750; CES-D: OR 1.863, 95% CI 1.036–3.350) (Table 3). However, when adjusting for depressive symptoms, the relationship between loneliness and cognitive impairment did not remain significant (Supplementary Table 7).

**TABLE 3:** Logistic regression between loneliness and sub-category of SNSB-C.

Variables	Attention		Language		Visuospatial function		Memory		Executive function	
	OR (95% CI)	*p*-Value	OR (95% CI)	*p*-Value	OR (95% CI)	*p*-Value	OR (95% CI)	*p*-Value	OR (95% CI)	*p*-Value
UCLA-LS	0.958 (0.591, 1.553)	0.862	1.489 (0.766, 2.897)	0.240	1.591 (1.029. 2.460)	0.037	1.601 (0.965, 2.655)	0.068	1.971 (1.036, 3.750)	0.039
CES-D-L	0.746 (0.488, 1.140)	0.176	1.662 (0.937, 2.950)	0.083	1.373 (0.935, 2.018)	0.106	1.577 (1.009, 2.466)	0.046	1.863 (1.036, 3.350)	0.038

CES-D-L, loneliness scale from Center for Epidemiologic Studies Depression Scale; MRI, magnetic resonance imaging; SNSB-C, Seoul Neuropsychological Screening Battery; UCLA-LS, UCLA-Loneliness Scale. Age, gender, education level, hypertension, diabetes, and CVD were adjusted.

### 3.3 Association between loneliness and brain MRI metrics

In the *T*-test analysis examining the relationship between loneliness and brain volumetrics, the group experiencing loneliness according to UCLA-LS had smaller volumes in cerebral white matter (MD 9.53, *p*-value 0.05), frontal white matter (right: MD 2.31, *p*-value 0.02; left: MD 2.3, *p*-value 0.02), left globus pallidus (MD 27.23, *p*-value 0.01), and left thalamus (MD 0.09, *p*-value 0.05), along with larger volumes of WMH (MD 3.09, *p*-value 0.01).

On the other hand, the group classified as lonely based on the loneliness item in CES-D had smaller volumes in total brain (MD 14.51, *p*-value 0.05), cerebral gray matter (MD 7.6, *p*-value 0.03), subcortical gray matter (MD 0.72, *p*-value 0.03), globus pallidus (right MD 24.43, *p*-value < 0.01; left: MD 27.73, *p*-value < 0.01), putamen (right MD 3.09, *p*-value 0.01; left: MD 0.11, *p*-value < 0.01), left thalamus (MD 0.10, *p*-value 0.02), and right hippocampus (MD 0.10, *p*-value < 0.01), along with a larger volume of WMH (MD 0.08, *p*-value 0.01) (Supplementary Table 5).

These trends were confirmed in linear regression analysis. In models adjusted for age, gender, education level, coexisting diseases, and intracranial volume, the group experiencing loneliness according to UCLA-LS had a 1.24 lower volume of the left frontal white matter (95% CI −2.37 ∼−0.12) and a 1.16 lower volume of the right frontal white matter (95% CI −2.31 ∼−0.00). The group classified as lonely based on CES-D-L had a 15.53 lower volume of the left globus pallidus (95% CI −30.13 ∼−0.93), a 0.07 lower volume of the left putamen (95% CI −0.12 ∼−0.02), and a 0.06 lower volume of the right putamen (95% CI −0.11 ∼−0.01). There was no statistically significant difference in WMH (Table 4). The relationship between loneliness and brain volume remained significant even after adjusting for depressive symptoms in the model (Supplementary Table 7).

**TABLE 4:** Linear regression for loneliness and MRI metrics.

MRI metrics	UCLA-LS		CES-D-L	
	β (95% CI)	*p*-Value	β (95% CI)	*p*-Value
Total brain volume	−3.20 (−9.02, 2.63)	0.28	−1.84 (−6.81, 3.12)	0.47
Cerebral white matter volume	−4.19 (−9.30, 0.92)	0.11	0.54 (−3.82, 4.91)	0.81
Cerebral gray matter volume	0.78 (−3.07, 4.63)	0.69	−2.09 (−5.37, 1.20)	0.21
Subcortical gray matter volume	0.22 (−0.52, 0.95)	0.56	−0.30 (−0.93, 0.32)	0.34
WMH volume (log)	0.05 (−0.02, 0.11)	0.14	0.03 (−0.03, 0.08)	0.32
Whole brain cortical thickness	0.00 (−0.01, 0.02)	0.97	0.00 (−0.02, 0.01)	0.74
**Left volume**				
Left frontal gray matter volume	−0.07 (−1.94, 1.80)	0.94	0.33 (−1.26, 1.93)	0.68
Left frontal white matter volume	−1.24 (−2.37, −0.12)	0.03	−0.21 (−1.18, 0.75)	0.66
Left hippocampus volume	0.01 (−0.04, 0.07)	0.63	0.01 (−0.04, 0.06)	0.70
Left caudate volume (log)	0.00 (−0.01, 0.01)	0.42	0.00 (−0.01, 0.00)	0.33
Left globus pallidus volume	−16.07 (−33.19, 1.06)	0.07	−15.53 (−30.13, −0.93)	0.04
Left putamen volume	−0.01 (−0.06, 0.05)	0.84	−0.07 (−0.12, −0.02)	0.01
**Right volume**				
Right frontal gray matter volume	0.26 (−1.59, 2.11)	0.78	0.71 (−0.87, 2.29)	0.38
Right frontal white matter volume	−1.16 (−2.31, 0.00)	0.05	−0.04 (−1.03, 0.95)	0.93
Right hippocampus volume	0.00 (−0.06, 0.06)	0.99	−0.01 (−0.06, 0.04)	0.71
Right caudate volume (log)	0.01 (0.00, 0.01)	0.26	0.00 (−0.01, 0.00)	0.28
Right globus pallidus volume	−6.80 (−23.17, 9.58)	0.42	−12.05 (−26.00, 1.90)	0.09
Right putamen volume	0.01 (−0.05, 0.07)	0.72	−0.06 (−0.11, −0.01)	0.02

CES-D-L, loneliness scale from Center for Epidemiologic Studies Depression Scale; MRI, magnetic resonance imaging; UCLA-LS, UCLA-Loneliness Scale. Age, gender, education level, hypertension, diabetes, CVD, and ICV volume were adjusted.

## 4 Discussion

The present study is the first, to our knowledge, to investigate loneliness, cognitive impairment, and structural brain changes in the Asian older adults. The analysis revealed that the group experiencing loneliness had a higher likelihood of cognitive impairment assessed by SNSB-C and K-MMSE-2, particularly showing differences in visuospatial abilities, memory, and executive function. From the perspective of structural brain changes, there were no differences in TBV, total gray matter volume, total white matter volume, or WMH. However, the volume of frontal white matter, left globus pallidus, and putamen was lower in the lonely group.

In the present study, two measures, UCLA-LS and CES-D-L, were used to define the group experiencing loneliness, and differences were observed in the association between loneliness and cognitive function, as well as brain volume, depending on the measure used. Therefore, we discuss the potential impact of loneliness measurement methods on the results. Generally, there are two main approaches to measure loneliness: using a scale and using a single-item measure. Scales such as UCLA-LS or the de Jong Gierveld scale are commonly employed, sometimes utilizing abbreviated versions with 8 or 3 items for respondent convenience. Scale-based measurements of loneliness, particularly UCLA-LS, often consist of indirect questions about loneliness. Loneliness is a sensitive and stigmatizing concept, leading to under-reporting when directly questioned, especially among male populations (Borys and Perlman, 1985; Osborn et al., 2018). Therefore, using a scale to measure loneliness can prevent this phenomenon. Single-item measures are also appropriate way to assess loneliness, and commonly used in large-scale surveys. Normative tests of various loneliness scales and single-item measures have shown that single-item measures share similar characteristics with scales (Mund et al., 2023). Like loneliness measured by scales, loneliness assessed by single-item measures also demonstrates associations with various health outcomes.

In the analysis of the current study, the loneliness group measured by UCLA-LS consisted of 132 individuals (16.8%), while the loneliness group measured by CES-D-L comprised 205 individuals (26.1%), indicating no apparent under-reporting phenomenon in the single item (Table 1). Several reasons may account for these results. Firstly, there could be issues related to the measurement process. Guidelines for loneliness measurement recommend administering scale-based measurements first to avoid interference between measurement tools conducting measurements using both scales and single items simultaneously (Snape and Martin, 2018; Ending Loneliness Together, 2021). However, in this study, the CES-D questionnaire was administered before the UCLA-LS. Consequently, respondents who indicated not feeling lonely in CES-D might have been influenced to respond less actively in the UCLA-LS survey as well. Additionally, in single-item measurements, it is assumed that questions about loneliness are conducted separately. However, the CES-D-L used in this study was derived from one item of the CES-D questionnaire, so other items in CES-D might have affected the response outcomes. Secondly, there could be differences in survey questions. While the single item recommended in the guidelines is “How often do you feel lonely?” and the CES-D-L item is “I felt lonely like being alone in the world,” including some indirect expressions, potentially resulting in a broader inclusion of individuals in the loneliness group than the question in the guidelines. Finally, most studies on loneliness measurement have been conducted primarily in Anglo-American culture, so responses to loneliness may be expressed differently in Asian culture (Ending Loneliness Together, 2021).

In the current study, there were 68 individuals classified as feeling lonely in both UCLA-LS and CES-D-L (Supplementary Table 2). The correlation analysis of the raw scores of UCLA-LS and CES-D-L showed a weak correlation of *r* = 0.33 (Supplementary Figure 1). Thus, while the classification through UCLA-LS and CES-D-L in this study showed some association, it is difficult to consider them as perfectly consistent, suggesting the possibility of two groups with different characteristics of loneliness. This difference may stem from the differences in the two surveys. UCLA-LS asks about one’s usual state, while CES-D assesses one’s state over the past week. Therefore, UCLA-LS may represent chronic loneliness, whereas CES-D-L may include situational loneliness related to recent events (Mund et al., 2023). In the general characteristics analysis of the two groups in this study, there were no differences in age or gender. However, the group measured by CES-D-L showed a higher prevalence of depressive symptoms (58.5%) compared to UCLA-LS, indicating that individuals feeling lonely in CES-D-L may experience greater depression than those in UCLA-LS (Supplementary Table 2).

The relationship between loneliness and cognitive function is well-established in previous research (Boss et al., 2015). According to existing studies, individuals experiencing loneliness have an odds ratio (OR) of 2.56 for developing dementia compared to those who do not feel lonely (Holwerda et al., 2014), and there is a decreased global cognitive function as well (Gow et al., 2007). The present study also confirmed that UCLA-LS and CES-D-L are related to the worse K-MMSE-2 and SNSB-C scores, aligning with previous research findings. Specifically, both UCLA-LS and CES-D-L showed the greatest association with executive function, which is often the first cognitive function to be impaired due to aging (Harada et al., 2013). Because the current study is cross-sectional, it is challenging to determine whether loneliness serves as an indicator or a risk factor for normal aging-related cognitive decline. However, recent longitudinal studies analyzing the relationship between loneliness and cognitive function suggest that loneliness is associated not only with baseline cognitive function but also with the rate of decline in cognitive function over time (Yin et al., 2019). Moreover, numerous biological mechanisms underlying the impact of loneliness on cognitive function have been elucidated, as will be discussed later. Therefore, it is likely that loneliness is more than just an indicator but rather a risk factor for cognitive decline.

While CES-D-L showed significant association only with K-MMSE-2, UCLA-LS demonstrated significant association only with SNSB-C (Table 2). This difference is believed to arise from the distinct characteristics of the cognitive assessment tools, K-MMSE-2 and SNSB-C. K-MMSE-2 is a validated screening tool for dementia but has a limited range of items compared to SNSB-C. Specifically, it has fewer items evaluating visuospatial function or executive function. Therefore, research has indicated that the MMSE has lower efficacy in assessing executive function compared to other assessment tools (Axelrod et al., 1992). On the other hand, SNSB-C includes validated assessment tools for five cognitive domains (attention, language, visuospatial function, memory, and executive function). Individuals classified as lonely by UCLA-LS exhibit decreased executive function and visuospatial function (Table 3), which may result in false-negative results in K-MMSE-2 due to its limited evaluation of these domains.

On the other hand, CES-D-L showed non-significant results with SNSB-C unlike UCLA-LS, and only significant results with K-MMSE-2. This could be attributed to higher attention scores among individuals classified as lonely group by CES-D-L, which may have offset the decreased results of memory and executive function. Further research is needed to explore the relationship between CES-D-L and attention scores. The association between CES-D-L and K-MMSE-2 remained significant even when using cutoff values adjusted for age and education level (Supplementary Table 4). When depressive symptoms were added to the model for both CES-D-L and UCLA-LS, cognitive impairment was not significant, indicating that depressive symptoms are a strong confounder in the relationship between loneliness and cognitive impairment (Supplementary Table 7). Some studies interpret loneliness as a risk factor for depression or as concurrent occurrences due to the same cause (Mund et al., 2023). Hence, there is a need for further research to explore the interaction between loneliness and depression more closely.

While empirical studies on loneliness and cognitive function have shown relatively consistent results, the relationship between loneliness and brain volume remains subject to debate, with varying findings across studies (Duffner et al., 2023). Examining previous research results, a study using Framingham study data (Salinas et al., 2022) found a statistically significant decrease in total brain volume (TBV) and WMH in individuals experiencing loneliness. However, a study using Rotterdam study data (Van Der Velpen et al., 2022) did not show significant differences in TBV, total gray matter volume, total white matter volume, and WMH. Similarly, in the results of the present study, there were no statistically significant differences in TBV, total gray matter volume, total white matter volume, and WMH, aligning with the findings from the Rotterdam study.

Several reasons could explain the inconsistency in results. Firstly, differences in variable definitions could be a contributing factor. The Framingham study defined loneliness as feeling lonely for 3 or more days a week, whereas the Rotterdam study defined it as feeling lonely for 1 or more days. While our study used the same criteria as the Framingham study, as described earlier, the loneliness-related questions in the CES-D may have been slightly different due to translation. Additionally, while the Framingham study used MRI results as a percentage of TCV, both the Rotterdam study and our study used raw values. Secondly, cultural differences across studies conducted in different regions may contribute to differences in subjective responses to loneliness, considering loneliness as a subjective reaction (Barreto et al., 2021). Lastly, variations in the criteria for excluding individuals with neurological abnormalities in each study could contribute to differences in the study populations and potentially lead to inconsistent results.

Lastly, the present study conducted an analysis of loneliness and regional brain volume. The results revealed that UCLA-LS was associated with a lower volume of right/left frontal white matter, while CES-D-L was associated with a lower volume of the left globus pallidus, left putamen, and right globus pallidus. The globus pallidus and putamen are regions that have not received much attention in previous cognitive function-related analyses. However, they, along with the frontal lobe, contribute to the formation of the putamen circuit and caudate circuit, mediating between the motor cortex and limbic system. Considering that executive function requires a speeded motor component (Hayden and Welsh-Bohmer, 2011) and that individuals experiencing loneliness showed a decrease in executive function, the lower volume of the frontal lobe, globus pallidus, and putamen may be related to the worse executive function.

Furthermore, the present study showed a lower frontal white matter volume even though total white matter volume did not decrease. Therefore, it is necessary to investigate the lower volume of specific regions of white matter rather than relying solely on total white matter volume. Additionally, even in cases where a decrease in total white matter volume is confirmed, there is a need to identify which specific regions of the brain experienced a decrease in white matter volume.

The mechanism by which loneliness influences cognitive function and brain structure has not been definitively established. However, considering the results showing a lower putamen and globus pallidus volume in lonely individuals in the present study, it is possible that frequent social activities stimulate the social reward circuit in these regions, preserving their function (Pierce and Péron, 2020). The nigrostriatal pathway is one of the dopamine pathways in the human brain, connecting the substantia nigra pars compacta (SNc) to the dorsal striatum (including the caudate nucleus and putamen). This pathway is responsible for motor function and reward-related cognition. While in the past, the reward circuit was thought to respond to monetary rewards, recent research has shown that it also responds to social rewards such as smiling faces. When dopaminergic neurons composing the nigrostriatal pathway degenerate, the volume of the striatum decreases, leading to reduced dopamine secretion. Engaging in appropriate social activities may mitigate these changes (Solomonov et al., 2023).

Moreover, striatal volume reduction is a characteristic of neurodegenerative diseases like Parkinson’s disease, which is also often accompanied by cognitive impairment. The exact mechanisms underlying cognitive impairment in Parkinson’s disease are not fully understood but are believed to involve degeneration of neurotransmitter systems and neuroinflammation (Aarsland et al., 2021). It is presumed that loneliness measured by CES-D-L may share similar mechanisms. However, loneliness measured by CES-D-L could also be considered an early indicator of Parkinson’s disease. Motor and cognitive impairments associated with Parkinson’s disease can disrupt social interactions and induce depression. However, cognitive impairments in Parkinson’s disease typically affect executive function and visuospatial function, while individuals identified as lonely by CES-D-L showed differences only in executive function. Therefore, further investigation is needed to determine whether CES-D-L serves as an early indicator of Parkinson’s disease or merely shares mechanisms with Parkinson’s disease and cognitive impairment. Finally, loneliness-induced inflammatory and cytotoxic responses might have contributed to neural damage affecting cognitive function (Kumar and Salinas, 2021).

Other components of social connection also show associations with cognitive function. For instance, the structural component of social connection, represented by social networks, has been studied in relation to brain volume. Unlike loneliness, which reflects subjective responses, social networks address objective social relationships. Research on the relationship between social networks and brain volume, much like loneliness, has focused on regions such as the amygdala volume, cortical thickness, and frontal lobe volume. Studies have shown that, in general, an increase in social network size is associated with an increase in the size of the amygdala (Bickart et al., 2011; Kanai et al., 2012; Von Der Heide et al., 2014). However, there have been conflicting results regarding cortical thickness (Bickart et al., 2011; Sherman et al., 2016; Sharifian et al., 2022) and frontal lobe volume (Powell et al., 2012; Von Der Heide et al., 2014). Nonetheless, findings suggest that as social network size increases, there is an increase in white matter microstructure in the frontal lobe (Noonan et al., 2018). With further research, it is anticipated that significant results will also emerge regarding the frontal lobe.

The present study has several strengths. First, it objectively evaluated age-related cognitive decline using well-established screening tools such as K-MMSE-2 and SNSB-C. Second, loneliness was measured using a systematic questionnaire with established validation, enhancing the accuracy of loneliness assessment and reducing misclassification by measurement tools. The present study also considered the differences between the two loneliness indicators used in the measurement. Lastly, the data analyzed in the current study were obtained from a long-operating local community cohort, collected by well-trained investigators. Therefore, the data has high completeness and reliability.

However, the present study also has limitations. First, being a cross-sectional study, it cannot determine the temporal sequence of loneliness, cognitive decline, and structural brain changes. To address this limitation, there is a need for longitudinal studies on loneliness and cognitive decline. Second, as a data-driven study analyzing multiple indicators simultaneously based on MRI data, chance differences could have occurred. When applying multiple comparison correction, statistically significant *p*-values are below 0.001, and using this threshold, there are no significant results in the MRI analysis. Nevertheless, the MRI indicators showing differences in the present study align with past research results. The simultaneous decrease in areas with similar functions, such as frontal white matter, globus pallidus, and putamen on both sides, provides a medical explanation, making it difficult to attribute the differences solely to chance. Thirdly, there were no physician diagnoses of dementia in the data we used. However, for the definition of cognitive impairment used in the analysis, clinical criteria were employed. Lastly, due to technical reasons, indicators like the prefrontal cortex and amygdala, which have previously shown high relevance, could not be included in the analysis.

The present study confirmed the association between loneliness and cognitive decline in individuals aged 55 and above. Additionally, through brain volume analysis, it was observed that the group experiencing loneliness exhibited a lower volume of the frontal cortex, putamen, and globus pallidus. This suggests a potential link to cognitive motor control or social reward circuits.

## Data availability statement

The data analyzed in this study is subject to the following licenses/restrictions: the data used in this analysis is sourced from the Institute of Genomic Cohort and is available upon request with appropriate IRB consent. Due to participant privacy concerns, the primary data cannot be provided directly. Requests to access these datasets should be directed to SK, kohhj@yonsei.ac.kr.

## Ethics statement

The studies involving humans were approved by the Institutional Review Board (IRB) of Wonju Severance Christian Hospital (IRB No. CR320120). The studies were conducted in accordance with the local legislation and institutional requirements. The participants provided their written informed consent to participate in this study.

## Author contributions

HL: Conceptualization, Data curation, Formal analysis, Investigation, Methodology, Visualization, Writing – original draft, Writing – review & editing. SY: Writing – review & editing. HC: Supervision, Writing – review & editing. GY: Writing – review & editing. SK: Conceptualization, Project administration, Supervision, Writing – review & editing.

## References

[B1] Aarsland D. Batzu L. Halliday G. Geurtsen G. Ballard C. Ray Chaudhuri K. (2021). Parkinson disease-associated cognitive impairment. *Nat. Rev. Dis. Prim.* 7:47. 10.3390/bs11050074 34210995

[B2] Axelrod B. Goldman R. Henry R. (1992). Sensitivity of the mini-mental state examination to frontal lobe dysfunction in normal aging. *J. Clin. Psychol.* 48 68–71. 10.1002/1097-4679(199201)48:1<68::aid-jclp2270480110>3.0.co;2-n 1556219

[B3] Barreto M. Victor C. Hammond C. Eccles A. Richins M. Qualter P. (2021). Loneliness around the world: Age, gender, and cultural differences in loneliness. *Pers. Individ. Differ.* 169:110066. 10.1016/j.paid.2020.110066 33536694 PMC7768187

[B4] Beutel M. Klein E. Brähler E. Reiner I. Jünger C. Michal M. (2017). Loneliness in the general population: Prevalence, determinants and relations to mental health. *BMC Psychiatry* 17:97. 10.1186/s12888-017-1262-x 28320380 PMC5359916

[B5] Bickart K. Wright C. Dautoff R. Dickerson B. Barrett L. (2011). Amygdala volume and social network size in humans. *Nat. Neurosci.* 14 163–164. 10.1038/nn.2724 21186358 PMC3079404

[B6] Borys S. Perlman D. (1985). Gender differences in loneliness. *Pers. Soc. Psychol. Bull.* 11 63–74. 10.3390/ijerph17249176 33302577 PMC7763056

[B7] Boss L. Kang D. Branson S. (2015). Loneliness and cognitive function in the older adult: A systematic review. *Int. Psychogeriatr.* 27 541–553. 10.1017/S1041610214002749 25554219

[B8] Cacioppo J. Patrick W. (2008). *Loneliness: Human nature and the need for social connection.* New York, NY: W W Norton & Co.

[B9] Campaign to End Loneliness (2024). *Our vision is that everyone can live a life free from chronic loneliness.* Available online at: https://www.campaigntoendloneliness.org/about-the-campaign/ (accessed March 20, 2024).

[B10] Coalition to End Social Isolation & Loneliness (2024). *Join the movement to end social isolation and loneliness.* Available online at: https://www.endsocialisolation.org/ (accessed March 20, 2024).

[B11] Committee on the Health and Medical Dimensions of Social Isolation and Loneliness in Older Adults, Board on Health Sciences Policy, Board on Behavioral, Cognitive, and Sensory Sciences, Health and Medicine Division, Division of Behavioral and Social Sciences and Education, and National Academies of Sciences, Engineering, and Medicine (2020). *Social isolation and loneliness in older adults: Opportunities for the health care system.* Washington, DC: National Academies Press. 32510896

[B12] Department for Digital, Culture, Media and Sport (2018). *A connected society: A strategy for tackling lonliness – laying the foundations for change.* London: Department for Digital, Culture, Media and Sport.

[B13] DiJulio B. Hamel L. Muñana C. Brodie M. (2018). *Loneliness and Social isolation in the United States, the United Kingdom, and Japan: An international surve.* San Francisco, CA: The Kaiser Family Foundation.

[B14] Duffner L. DeJong N. Jansen J. Backes W. De Vugt M. Deckers K. (2023). Associations between social health factors, cognitive activity and neurostructural markers for brain health – A systematic literature review and meta-analysis. *Ageing Res. Rev.* 89:101986. 10.1016/j.arr.2023.101986 37356551

[B15] Ending Loneliness Together (2021). *A guide to measuring loneliness for community organisations.* Sydney, NSW: Ending Loneliness Together.

[B16] Ending loneliness together (2024). *Imagine a world where everyone feels a sense of connection and belonging.* Available online at: https://endingloneliness.com.au/ (accessed March 20, 2024).

[B17] Gow A. Pattie A. Whiteman M. Whalley L. Deary I. (2007). Social support and successful aging: Investigating the relationships between lifetime cognitive change and life satisfaction. *J. Individ. Differ.* 28 103–115. 10.1027/1614-0001.28.3.103

[B18] Harada C. Natelson Love M. Triebel K. (2013). Normal cognitive aging. *Clin. Geriatr. Med.* 29 737–752. 10.1016/j.cger.2013.07.002 24094294 PMC4015335

[B19] Hayden K. Welsh-Bohmer K. (2011). “Epidemiology of cognitive aging and Alzheimer’s disease: Contributions of the cache county Utah study of memory, Health and Aging,” in *Behavioral neurobiology of aging*, eds Pardon M. Bondi M. (Berlin: Springer). 10.1007/7854_2011_152 21809193

[B20] Henschel L. Conjeti S. Estrada S. Diers K. Fischl B. Reuter M. (2020). FastSurfer – A fast and accurate deep learning based neuroimaging pipeline. *Neuroimage* 219:117012. 10.1016/j.neuroimage.2020.117012 32526386 PMC7898243

[B21] Holt-Lunstad J. (2018). Why social relationships are important for physical health: A systems approach to understanding and modifying risk and protection. *Annu. Rev. Psychol.* 69 437–458. 10.1146/annurev-psych-122216-011902 29035688

[B22] Holt-Lunstad J. (2022). Social connection as a public health issue: The evidence and a systemic framework for prioritizing the “social” in social determinants of health. *Annu. Rev. Public Health* 43 193–213. 10.1146/annurev-publhealth-052020-110732 35021021

[B23] Holt-Lunstad J. Smith T. Baker M. Harris T. Stephenson D. (2015). Loneliness and social isolation as risk factors for mortality: A meta-analytic review. *Perspect. Psychol. Sci.* 10 227–237. 10.1177/1745691614568352 25910392

[B24] Holwerda T. Deeg D. Beekman A. Van Tilburg T. Stek M. Jonker C. (2014). Feelings of loneliness, but not social isolation, predict dementia onset: Results from the Amsterdam study of the elderly (AMSTEL). *J. Neurol. Neurosurg. Psychiatry* 85 135–142. 10.1136/jnnp-2012-302755 23232034

[B25] Jahng S. Na D. Kang Y. (2015). Constructing a composite score for the seoul neuropsychological screening battery-core. *Dement. Neurocogn. Disord.* 14:137. 10.12779/dnd.2023.22.1.1 36814700 PMC9939572

[B26] Kanai R. Bahrami B. Roylance R. Rees G. (2012). Online social network size is reflected in human brain structure. *Proc. R. Soc. B Biol. Sci.* 279 1327–1334. 10.1098/rspb.2011.1959 22012980 PMC3282379

[B27] Kim Y. Han B. The KoGES group. (2017). Cohort profile: The Korean genome and epidemiology study (KoGES) consortium. *Int. J. Epidemiol.* 46 e20–e20. 10.1093/ije/dyv316 27085081 PMC5837648

[B28] Kumar A. Salinas J. (2021). The long-term public health impact of social distancing on brain health: Topical review. *Int. J. Environ. Res. Public Health* 18:7307. 10.3390/ijerph18147307 34299756 PMC8305633

[B29] Lara E. Martín-María N. De La Torre-Luque A. Koyanagi A. Vancampfort D. Izquierdo A. (2019). Does loneliness contribute to mild cognitive impairment and dementia? A systematic review and meta-analysis of longitudinal studies. *Ageing Res. Rev.* 52 7–16. 10.1016/j.arr.2019.03.002 30914351

[B30] Lepage C. Wagstyl K. Jung B. Seidlitz J. Sponheim C. Ungerleider L. (2021). CIVET-Macaque: An automated pipeline for MRI-based cortical surface generation and cortical thickness in macaques. *Neuroimage* 227:117622. 10.1016/j.neuroimage.2020.117622 33301944 PMC7615896

[B31] Livingston G. Huntley J. Sommerlad A. Ames D. Ballard C. Banerjee S. (2020). Dementia prevention, intervention, and care: 2020 report of the Lancet Commission. *Lancet* 396 413–446. 10.1016/S0140-6736(20)30367-6 32738937 PMC7392084

[B32] Lobo A. LóPez-Antón R. De-La-CÁmara C. Quintanilla M. Campayo A. Saz P. (2008). Non-cognitive psychopathological symptoms associated with incident mild cognitive impairment and dementia, Alzheimer’s type. *Neurotox. Res.* 14 263–272. 10.1007/BF03033815 19073431

[B33] Lykes V. Kemmelmeier M. (2014). What predicts loneliness? Cultural difference between individualistic and collectivistic societies in Europe. *J. Cross Cult. Psychol.* 45 468–490.

[B34] Mund M. Maes M. Drewke P. Gutzeit A. Jaki I. Qualter P. (2023). Would the real loneliness please stand up? The validity of loneliness scores and the reliability of single-item scores. *Assessment* 30 1226–1248. 10.1177/10731911221077227 35246009 PMC10149889

[B35] Murthy V. (2017). *Work and the loneliness epidemic.* Boston, MA: Harvard Business Publishing, 26.

[B36] Noonan M. Mars R. Sallet J. Dunbar R. Fellows L. (2018). The structural and functional brain networks that support human social networks. *Behav. Brain Res.* 355 12–23. 29471028 10.1016/j.bbr.2018.02.019 PMC6152579

[B37] Osborn E. Martin G. Cochrane A. Hassell C. (2018). *Testing of loneliness questions in surveys.* Newport: Office for National Statistics.

[B38] Perissinotto C. Stijacic Cenzer I. Covinsky K. (2012). Loneliness in older persons: A predictor of functional decline and death. *Arch. Intern Med.* 172 1078–1083. 10.1001/archinternmed.2012.1993 22710744 PMC4383762

[B39] Perlman D. Peplau L. A. (1981). *Toward a social psychology of loneliness.* London: Academic Press.

[B40] Pierce J. Péron J. (2020). The basal ganglia and the cerebellum in human emotion. *Soc. Cogn. Affect. Neurosci.* 15 599–613. 32507876 10.1093/scan/nsaa076 PMC7328022

[B41] Powell J. Lewis P. Roberts N. García-Fiñana M. Dunbar R. (2012). Orbital prefrontal cortex volume predicts social network size: An imaging study of individual differences in humans. *Proc. R. Soc. B Biol. Sci.* 279 2157–2162. 10.1098/rspb.2011.2574 22298855 PMC3321718

[B42] Prince M. Ali G. Guerchet M. Prina A. Albanese E. Wu Y. (2016). Recent global trends in the prevalence and incidence of dementia, and survival with dementia. *Alzheimers Res. Ther.* 8:23. 10.1186/s13195-016-0188-8 PMC4967299 27473681

[B43] Russell D. (1996). UCLA loneliness scale (version 3): Reliability, validity, and factor structure. *J. Pers. Assess.* 66 20–40. 10.1207/s15327752jpa6601_2 8576833

[B44] Salinas J. Beiser A. Samra J. O’Donnell A. DeCarli C. Gonzales M. (2022). Association of loneliness with 10-year dementia risk and early markers of vulnerability for neurocognitive decline. *Neurology* 98 e1337–e1348. 35131906 10.1212/WNL.0000000000200039 PMC8967424

[B45] Sharifian N. Zaheed A. Morris E. Sol K. Manly J. Schupf N. (2022). Social network characteristics moderate associations between cortical thickness and cognitive functioning in older adults. *Alzheimers Dement.* 18 339–347. 10.1002/alz.12383 34002926 PMC8599522

[B46] Sherman S. Cheng Y. Fingerman K. Schnyer D. (2016). Social support, stress and the aging brain. *Soc. Cogn. Affect. Neurosci.* 11 1050–1058. 26060327 10.1093/scan/nsv071 PMC4927026

[B47] Snape D. Martin G. (2018). *Measuring loneliness: guidance for use of the national indicators on surveys.* Newport: Office for National Statistics.

[B48] Solomonov N. Victoria L. Lyons K. Phan D. Alexopoulos G. Gunning F. (2023). Social reward processing in depressed and healthy individuals across the lifespan: A systematic review and a preliminary coordinate-based meta-analysis of fMRI studies. *Behav. Brain Res.* 454:114632. 10.1016/j.bbr.2023.114632 37598904 PMC10557626

[B49] Surkalim D. Luo M. Eres R. Gebel K. Van Buskirk J. Bauman A. (2022). The prevalence of loneliness across 113 countries: Systematic review and meta-analysis. *BMJ* 9:e067068. 10.1136/bmj-2021-067068 35140066 PMC8826180

[B50] Tomohiro Osaki (2021). *As suicides rise amid the pandemic, japan takes steps to tackle loneliness.* Tokyo: TOMOHIRO OSAKI.

[B51] Valtorta N. Kanaan M. Gilbody S. Ronzi S. Hanratty B. (2016). Loneliness and social isolation as risk factors for coronary heart disease and stroke: Systematic review and meta-analysis of longitudinal observational studies. *Heart* 102 1009–1016. 10.1136/heartjnl-2015-308790 27091846 PMC4941172

[B52] Van Der Velpen I. Melis R. Perry M. Vernooij-Dassen M. Ikram M. Vernooij M. (2022). Social health is associated with structural brain changes in older adults: The Rotterdam study. *Biol. Psychiatry Cogn. Neurosci. Neuroimaging* 7 659–668. 10.1016/j.bpsc.2021.01.009 33549803

[B53] Von Der Heide R. Vyas G. Olson I. (2014). The social network-network: Size is predicted by brain structure and function in the amygdala and paralimbic regions. *Soc. Cogn. Affect. Neurosci.* 9 1962–1972. 10.1093/scan/nsu009 24493846 PMC4249478

[B54] Weiner M. Veitch D. Aisen P. Beckett L. Cairns N. Cedarbaum J. (2015). Impact of the Alzheimer’s disease neuroimaging initiative, 2004 to 2014. *Alzheimers Dement.* 11 865–884. 10.1016/j.jalz.2015.04.005 26194320 PMC4659407

[B55] World Health Organization (2021). *Social isolation and loneliness among older people: Advocacy brief.* Geneva: World Health Organization.

[B56] Yin J. Lassale C. Steptoe A. Cadar D. (2019). Exploring the bidirectional associations between loneliness and cognitive functioning over 10 years: The English longitudinal study of ageing. *Int. J. Epidemiol.* 48 1937–1948. 10.1093/ije/dyz085 31056641 PMC6929532

