# Supplementary material for: Association between loneliness and cognitive function, and brain volume in community-dwelling elderly
Source: Front Aging Neurosci. 2024 Apr 29;16:1389476. doi: 10.3389/fnagi.2024.1389476 (PMC11089178; doi:10.3389/fnagi.2024.1389476)
Supplement: Supplementary file 1 [file Data_Sheet_1.docx]

Supplementary Material for Association between loneliness and cognitive function, and brain volume in community-dwelling elderly

# Supplementary Data

## Brain MRI sample parameters

T1: 176x240x256 (1,1,1) [TR=2.3s, TE=2.96ms]

FLAIR: 160x256x256 (1.2,1,1) [TR=4.8s, TE=441ms]

T2: 256x256x44 (0.859, 0.859, 4) [TR=650ms, TE=20ms]

fMRI: 64x64x48 *197 (3.437,3.438,3.4) [TR=3s, TE=30ms]

DTI: 116x116x80 *33 (2,2,2) [TR=9.6s, TE=82ms]

Field map: 64x64x55 (3.625,3.625,3) [TR=600ms, TE=7.38ms]

# Supplementary Tables

## Supplementary Table 1. Brain MRI protocol

|  | T1 | T2 | DTI | fMRI | FLAIR |
| --- | --- | --- | --- | --- | --- |
| Resolution | 1x1x1 mm | 0.8x0.8x4 mm | 2x2x2 mm | 2.4x2.4x2.4 mm | 1.05x1x1 mm |
| Field-of-View | 208x256x256 matrix | 256x256x44 matrix | 104x104x72 matrix | 88x88x88 matrix | 192x256x256 matrix |
| Duration | 5 min | 4 min | 7 min (including 36 seconds phase-encoding reversed data) | 6 min (490 time point) | 6 min |
| Options | 3d MPRAGE, Sagittal, In-plane acceleration iPAT=2, prescan-normalize | Axia T2 star / GRE, TR=650ms, TE=20ms, filter=2D Distortion Corr, Prescan Normalize, Elliptical filter | phase-encoding reversed data 5xb=0(+3xb=0blipreversed), 50xb=1000, 50xb=2000 SE-EPI withx3 multislice acceleration,no iPAT, fat saturation | TR:0.735s TE:39ms GE-EPI withx8 multislice accleration,no iPAT,  flipangle 52°, fat saturation | 3D SPACE,  sagittal, in-plane acceleration iPAT=2, partialFourier=7/8, fatsaturation, elliptical k-space scanning,  prescan-normalise |

## Supplementary Table 2. Baseline characteristics by loneliness measurement

| Variables | Total (n=785) | Lonely group by UCLA-LS (n=132) | Lonely group by CES-D-L (n=205) |
| --- | --- | --- | --- |
| Age, mean±SD | 67.3±6.3 | 68.9±6.2 | 68.5±5.9 |
| Sex, n(%) |  |  |  |
| Male | 292 (37.2) | 46 (34.8) | 71 (34.6) |
| Female | 493 (62.8) | 86 (65.2) | 134 (65.4) |
| Education, n(%) |  |  |  |
| Under high school | 358 (45.6) | 77 (58.3) | 115 (56.1) |
| High school | 229 (29.2) | 30 (22.7) | 46 (22.4) |
| Upper college | 198 (25.1) | 25 (18.9) | 44 (21.5) |
| Comorbidities, n(%) |  |  |  |
| Hypertension | 429 (54.6) | 71 (53.8) | 113 (55.1) |
| Diabetes | 179 (22.8) | 37 (28.0) | 58 (28.3) |
| Cardiovascular disease | 93 (11.8) | 19 (14.4) | 28 (13.7) |
| Depressive symptom | 163 (20.8) | 65 (49.2) | 120 (58.5) |
| Lonely group vs. not lonely group |  |  |  |
| Based on UCLA-LS, n(%) | 132 (16.8) | - | 68 (33.2) |
| Based on CES-D-L, n(%) | 205 (26.1) | 68 (51.5) | - |
| Cognitive impairment (based on K-MMSE-2), n(%) | 35 (4.5) | 13 (9.8) | 17 (8.3) |
| Cognitive impairment (based on SNSB-C), n(%) |  |  |  |
| Attention | 164 (20.9) | 25 (18.9) | 34 (16.6) |
| Language | 60 (7.6) | 13 (9.8) | 21 (10.2) |
| Visuospatial function | 169 (21.5) | 37 (28.0) | 52 (25.4) |
| Memory | 109 (13.9) | 25 (18.9) | 37 (18.0) |
| Frontal/Executive function | 53 (6.8) | 13 (9.8) | 21 (10.2) |
| Total | 93 (11.8) | 26 (19.7) | 28 (13.7) |
| Total cerebral volume, mean±SD | 979.9±89.16 | 968.66±89.49 | 969.18±89.83 |
| Cerebral white matter volume, mean±SD | 156.41±51.97 | 448.48±48.94 | 451.84±52.07 |
| Cerebral gray matter volume, mean±SD | 193.62±43.15 | 490.51±48.76 | 488.00±43.64 |
| Subcortical gray matter volume, mean±SD | 29.88±4.15 | 29.68±4.43 | 29.34±4.06 |
| WMH volume, mean±SD | 3.43±0.39 | 3.52±0.43 | 3.49±0.42 |

* CES-D-L: Lonelieness scale from Center for Epidemiologic Studies Depression Scale; K-MMSE-2: Korean version of Mini-Mental State Examination 2; MRI: Magnetic Resonance Imaging; SD: Standard Deviation; SNSB-C: Seoul Neuropsychological Screening Battery; UCLA-LS: UCLA-Loneliness Scale

## Supplementary Table 3. Univariate analysis for loneliness and neuropsychological test

| Cognitive impairment | UCLA-LS | | | CES-D-L | | |
| --- | --- | --- | --- | --- | --- | --- |
|  | Not lonely | Lonely | p-value | Not lonely | Lonely | p-value |
| K-MMSE-2 |  |  | < 0.01 |  |  | < 0.01 |
| No | 631 | 119 |  | 562 | 188 |  |
| Yes | 22 | 13 |  | 18 | 17 |  |
| SNSB-C: Attention |  |  | 0.63 |  |  | 0.10 |
| No | 514 | 107 |  | 450 | 171 |  |
| Yes | 139 | 25 |  | 130 | 34 |  |
| SNSB-C: Language |  |  | 0.39 |  |  | 0.14 |
| No | 606 | 119 |  | 541 | 184 |  |
| Yes | 47 | 13 |  | 39 | 21 |  |
| SNSB-C: Visuospatial function |  |  | 0.06 |  |  | 0.15 |
| No | 521 | 95 |  | 463 | 153 |  |
| Yes | 132 | 37 |  | 117 | 52 |  |
| SNSB-C: memory |  |  | 0.09 |  |  | 0.06 |
| No | 569 | 107 |  | 508 | 168 |  |
| Yes | 84 | 25 |  | 72 | 37 |  |
| SNSB-C: Executive function |  |  | 0.03 |  |  | 0.03 |
| No | 615 | 117 |  | 548 | 184 |  |
| Yes | 38 | 15 |  | 32 | 21 |  |
| SNSB-C: Total |  |  | < 0.01 |  |  | 0.42 |
| No | 586 | 106 |  | 515 | 177 |  |
| Yes | 67 | 26 |  | 65 | 28 |  |

* CES-D-L: Loneliness scale from Center for Epidemiologic Studies Depression Scale; K-MMSE-2: Korean version of Mini-Mental State Examination 2; SNSB-C: Seoul Neuropsychological Screening Battery; UCLA-LS: UCLA-Loneliness Scale

## Supplementary Table 4. Logistic regression for Loneliness and K-MMSE-2

| Variables | Cutoff: < 1 SD | | | | Cutoff: < 2 SD | | | |
| --- | --- | --- | --- | --- | --- | --- | --- | --- |
|  | Univariate | | Multivariate | | Univariate | | Multivariate | |
|  | OR (95% CI) | p-value | OR (95% CI) | p-value | OR (95% CI) | p-value | OR (95% CI) | p-value |
| UCLA-LS | 1.423 (0.880, 2.301) | 0.150 | 1.512 (0.924, 2.474) | 0.100 | 1.606 (0.672, 3.841) | 0.287 | 1.569 (0.636, 3.871) | 0.328 |
| CES-D-L | 1.007 (0.649, 1.562) | 0.975 | 1.048 (0.669, 1.642) | 0.838 | 2.387 (1.127, 5.052) | 0.023 | 2.284 (1.057, 4.932) | 0.036 |

* CES-D-L: Loneliness scale from Center for Epidemiologic Studies Depression Scale; K-MMSE-2: Korean version of Mini-Mental State Examination 2; UCLA-LS: UCLA-Loneliness Scale

## Supplementary Table 5. Univariate analysis for Loneliness and MRI metrics

| MRI metrics | UCLA-LS | | | CES-D-L | | |
| --- | --- | --- | --- | --- | --- | --- |
|  | Not lonely | Lonely | p-value | Not lonely | Lonely | p-value |
| Intracranial volume | 1330.25 ± 118.35 | 1326.03 ± 117.95 | 0.71 | 1331.89 ± 117.02 | 1322.88 ± 121.61 | 0.36 |
| Total brain volume | 982.17 ± 88.99 | 968.66 ± 89.49 | 0.12 | 983.69 ± 88.69 | 969.18 ± 89.83 | 0.05 |
| Cerebral white matter volume | 458.01 ± 52.45 | 448.48 ± 48.94 | 0.05 | 458.02 ± 51.88 | 451.84 ± 52.07 | 0.14 |
| Cerebral graymatter volume | 494.25 ± 41.94 | 490.51 ± 48.76 | 0.41 | 495.60 ± 42.84 | 488.00 ± 43.64 | 0.03 |
| Subcortical gray matter volume | 29.92 ± 4.09 | 29.68 ± 4.43 | 0.57 | 30.06 ± 4.17 | 29.34 ± 4.06 | 0.03 |
| 3rd ventricle volume (log) | 3.37 ± 0.15 | 3.42 ± 0.14 | 0.00 | 3.37 ± 0.16 | 3.40 ± 0.14 | 0.02 |
| 4th ventricle volume (log) | 3.19 ± 0.17 | 3.22 ± 0.16 | 0.13 | 3.19 ± 0.17 | 3.21 ± 0.16 | 0.08 |
| Brainstem volume | 32.36 ± 3.75 | 31.89 ± 3.47 | 0.16 | 32.37 ± 3.68 | 32.02 ± 3.77 | 0.24 |
| CSF volume (log) | 5.07 ± 0.10 | 5.10 ± 0.10 | 0.01 | 5.07 ± 0.10 | 5.09 ± 0.10 | 0.04 |
| Extracerebral CSF volume | 484.98 ± 87.28 | 488.17 ± 95.33 | 0.72 | 485.99 ± 87.86 | 484.16 ± 90.98 | 0.80 |
| WMH volume (log) | 3.41 ± 0.38 | 3.52 ± 0.43 | 0.01 | 3.41 ± 0.37 | 3.49 ± 0.42 | 0.03 |
| Deep WMH volume (log) | 3.16 ± 0.36 | 3.24 ± 0.45 | 0.06 | 3.16 ± 0.36 | 3.23 ± 0.43 | 0.05 |
| Periventricula hyperintensity volume (log) | 2.94 ± 0.57 | 3.10 ± 0.54 | 0.00 | 2.94 ± 0.58 | 3.05 ± 0.52 | 0.01 |
| Whole brain cortical thickness | 3.17 ± 0.10 | 3.15 ± 0.11 | 0.25 | 3.17 ± 0.10 | 3.15 ± 0.10 | 0.06 |
| Left cortical thickness | 3.14 ± 0.10 | 3.13 ± 0.11 | 0.60 | 3.14 ± 0.10 | 3.13 ± 0.10 | 0.16 |
| Right cortical thickness | 3.19 ± 0.10 | 3.17 ± 0.11 | 0.08 | 3.19 ± 0.10 | 3.17 ± 0.10 | 0.02 |
| Left volume |  |  |  |  |  |  |
| Left caudate volume (log) | 3.64 ± 0.05 | 3.65 ± 0.06 | 0.31 | 3.64 ± 0.05 | 3.64 ± 0.06 | 0.53 |
| Left cerebellum volume | 61.50 ± 5.93 | 60.83 ± 5.65 | 0.22 | 61.62 ± 6.00 | 60.74 ± 5.51 | 0.05 |
| Left fornix volume | 539.54 ± 86.93 | 529.98 ± 75.99 | 0.20 | 540.40 ± 86.37 | 530.97 ± 81.68 | 0.16 |
| Left frontal gray matter volume | 126.40 ± 13.05 | 125.89 ± 13.79 | 0.70 | 126.44 ± 13.48 | 125.97 ± 12.29 | 0.65 |
| Left frontal white matter volume | 91.75 ± 10.87 | 89.45 ± 10.35 | 0.02 | 91.77 ± 10.84 | 90.20 ± 10.69 | 0.07 |
| Left globus pallidus volume | 887.38 ± 104.42 | 860.15 ± 103.62 | 0.01 | 890.04 ± 105.44 | 862.31 ± 100.09 | 0.00 |
| Left hippocampus volume | 3.02 ± 0.33 | 2.98 ± 0.36 | 0.20 | 3.02 ± 0.34 | 2.98 ± 0.34 | 0.12 |
| Left lateral ventricle volume (log) | 4.07 ± 0.22 | 4.13 ± 0.22 | 0.00 | 4.07 ± 0.22 | 4.10 ± 0.22 | 0.11 |
| Left occipital gray matter volume | 38.61 ± 5.26 | 39.02 ± 5.50 | 0.44 | 38.52 ± 5.45 | 39.14 ± 4.82 | 0.12 |
| Left occipital white matter volume | 22.19 ± 3.13 | 22.13 ± 2.90 | 0.84 | 22.17 ± 3.08 | 22.22 ± 3.14 | 0.84 |
| Left parietal gray matter volume | 69.28 ± 8.75 | 68.90 ± 9.79 | 0.68 | 68.97 ± 8.96 | 69.91 ± 8.82 | 0.19 |
| Left parietal white matter volume (log) | 4.71 ± 0.05 | 4.70 ± 0.05 | 0.08 | 4.71 ± 0.05 | 4.70 ± 0.05 | 0.29 |
| Left putamen volume | 3.80 ± 0.39 | 3.75 ± 0.39 | 0.21 | 3.82 ± 0.40 | 3.71 ± 0.35 | 0.00 |
| Left subthalamic nucleus volume | 43.85 ± 7.50 | 42.77 ± 7.10 | 0.12 | 43.94 ± 7.44 | 42.91 ± 7.41 | 0.09 |
| Left temporal gray matter volume | 91.50 ± 9.21 | 91.36 ± 10.21 | 0.89 | 91.65 ± 9.47 | 90.98 ± 9.13 | 0.38 |
| Left temporal white matter volume (log) | 4.69 ± 0.05 | 4.68 ± 0.05 | 0.22 | 4.69 ± 0.05 | 4.68 ± 0.05 | 0.36 |
| Left thalamus volume | 6.64 ± 0.53 | 6.55 ± 0.52 | 0.05 | 6.65 ± 0.53 | 6.55 ± 0.55 | 0.02 |
| Right volume |  |  |  |  |  |  |
| Right caudate volume (log) | 3.65 ± 0.06 | 3.66 ± 0.07 | 0.16 | 3.65 ± 0.06 | 3.65 ± 0.06 | 0.66 |
| Right cerebellum volume | 62.88 ± 6.11 | 62.33 ± 5.66 | 0.31 | 62.99 ± 6.11 | 62.21 ± 5.82 | 0.10 |
| Right fornix volume | 533.15 ± 80.94 | 514.11 ± 74.04 | 0.01 | 533.76 ± 80.41 | 519.16 ± 78.38 | 0.02 |
| Right frontal gray matter volume | 126.07 ± 13.08 | 125.89 ± 13.66 | 0.89 | 126.06 ± 13.46 | 125.97 ± 12.33 | 0.93 |
| Right frontal white matter volume | 91.27 ± 11.11 | 88.96 ± 10.52 | 0.02 | 91.27 ± 11.08 | 89.79 ± 10.87 | 0.10 |
| Right globus pallidus volume | 851.33 ± 100.69 | 832.91 ± 100.11 | 0.06 | 854.61 ± 100.36 | 830.18 ± 99.96 | 0.00 |
| Right hippocampus volume | 3.20 ± 0.36 | 3.14 ± 0.37 | 0.07 | 3.21 ± 0.35 | 3.14 ± 0.37 | 0.02 |
| Right lateral ventricle volume (log) | 3.99 ± 0.23 | 4.07 ± 0.24 | 0.00 | 3.99 ± 0.23 | 4.04 ± 0.23 | 0.01 |
| Right occipital gray matter volume | 39.93 ± 5.22 | 40.55 ± 5.81 | 0.25 | 39.91 ± 5.43 | 40.36 ± 5.01 | 0.28 |
| Right occipital white matter volume (log) | 4.36 ± 0.06 | 4.36 ± 0.06 | 0.94 | 4.36 ± 0.06 | 4.36 ± 0.06 | 0.83 |
| Right parietal gray matter volume | 67.61 ± 7.89 | 66.97 ± 8.98 | 0.45 | 67.28 ± 8.19 | 68.14 ± 7.75 | 0.18 |
| Right parietal white matter volume (log) | 4.71 ± 0.05 | 4.70 ± 0.05 | 0.07 | 4.71 ± 0.05 | 4.71 ± 0.05 | 0.42 |
| Right putamen volume | 3.90 ± 0.39 | 3.86 ± 0.42 | 0.36 | 3.92 ± 0.40 | 3.82 ± 0.37 | 0.00 |
| Right subthalamic nucleus volume | 44.7 ± 7.39 | 44.49 ± 7.58 | 0.77 | 44.95 ± 7.27 | 43.85 ± 7.79 | 0.08 |
| Right temporal gray matter volume | 93.62 ± 9.00 | 93.12 ± 10.78 | 0.62 | 93.8 ± 9.32 | 92.78 ± 9.31 | 0.18 |
| Right temporal white matter volume (log) | 4.70 ± 0.05 | 4.70 ± 0.05 | 0.11 | 4.70 ± 0.05 | 4.70 ± 0.05 | 0.39 |
| Right thalamus volume | 6.69 ± 0.53 | 6.61 ± 0.52 | 0.11 | 6.70 ± 0.52 | 6.62 ± 0.55 | 0.06 |

* CES-D-L: Loneliness scale from Center for Epidemiologic Studies Depression Scale; MRI: Magnetic Resonance Imaging; UCLA-LS: UCLA-Loneliness Scale

## Supplementary Table 6. Linear regression for loneliness and MRI metrics

| MRI metrics | UCLA-LS | | | |  | CES-D-L | | | |
| --- | --- | --- | --- | --- | --- | --- | --- | --- | --- |
|  | β (95% CI) | | | p-value |  | β (95% CI) | | | p-value |
| Total brain volume | -3.20 (-9.02, 2.63) | | | 0.28 |  | -1.84 (-6.81, 3.12) | | | 0.47 |
| Cerebral white matter volume | -419 (-9.30, 0.92) | | | 0.11 |  | 0.54 (-3.82, 4.91) | | | 0.81 |
| Cerebral gray matter volume | 0.78 (-3.07, 4.63) | | | 0.69 |  | -2.09 (-5.37, 1.20) | | | 0.21 |
| Subcortical gray matter volume | 0.22 (-0.52, 0.95) | | | 0.56 |  | -0.30 (-0.93, 0.32) | | | 0.34 |
| 3rd ventricle volume (log) | 0.03 (0.00, 0.05) | | | 0.03 |  | 0.01 (-0.01, 0.03) | | | 0.17 |
| 4th ventricle volume (log) | 0.01 (-0.02, 0.04) | | | 0.50 |  | 0.01 (-0.01, 0.04) | | | 0.31 |
| Brainstem volume | -0.28 (-0.75, 0.20) | | | 0.26 |  | -0.05 (-0.46, 0.36) | | | 0.81 |
| CSF volume (log) | 0.01 (0.00, 0.02) | | | 0.08 |  | 0.01 (-0.00, 0.02) | | | 0.14 |
| Extracerebral CSF volume | -0.42 (-12.51, 11.68) | | | 0.95 |  | -2.64 (-12.96, 7.68) | | | 0.62 |
| WMH volume (log) | 0.05 (-0.02, 0.11) | | | 0.14 |  | 0.03 (-0.03, 0.08) | | | 0.32 |
| Deep WMH volume (log) | 0.04 (-0.02, 0.11) | | | 0.21 |  | 0.04 (-0.02, 0.09) | | | 0.22 |
| Periventricula hyperintensity volume (log) | 0.06 (-0.03, 0.15) | | | 0.18 |  | 0.04 (-0.04, 0.11) | | | 0.37 |
| Whole brain cortical thickness | 0.00 (-0.01, 0.02) | | | 0.97 |  | 0.00 (-0.02, 0.01) | | | 0.74 |
| Left cortical thickness | 0.01 (-0.01, 0.02) | | | 0.46 |  | 0.00 (-0.01, 0.01) | | | 0.91 |
| Right cortical thickness | -0.01 (-0.02, 0.01) | | | 0.51 |  | -0.01 (-0.02, 0.01) | | | 0.44 |
| Left volume |  |  |  |  |  |  |  |  |  |
| Left caudate volume (log) | 0.00 (-0.01, 0.01) | | | 0.42 |  | 0.00 (-0.01, 0.00) | | | 0.33 |
| Left cerebellum volume | -0.10 (-0.91, 0.71) | | | 0.81 |  | -0.22 (-0.91, 0.47) | | | 0.53 |
| Left fornix volume | 1.57 (-12.22, 15.35) | | | 0.82 |  | 2.02 (-9.74, 13.78) | | | 0.74 |
| Left frontal gray matter volume | -0.07 (-1.94, 1.80) | | | 0.94 |  | 0.33 (-1.26, 1.93) | | | 0.68 |
| Left frontal white matter volume | -1.24 (-2.37, -0.12) | | | 0.03 |  | -0.21 (-1.18, 0.75) | | | 0.66 |
| Left globus pallidus volume | -16.07 (-33.19, 1.06) | | | 0.07 |  | -15.53 (-30.13, -0.93) | | | 0.04 |
| Left hippocampus volume | 0.01 (-0.04, 0.07) | | | 0.63 |  | 0.01 (-0.04, 0.06) | | | 0.70 |
| Left lateral ventricle volume (log) | 0.03 (0.00, 0.06) | | | 0.07 |  | 0.00 (-0.02, 0.03) | | | 0.74 |
| Left occipital gray matter volume | 0.26 (-0.56, 1.09) | | | 0.53 |  | 0.63 (-0.07, 1.33) | | | 0.08 |
| Left occipital white matter volume | 0.04 (-0.42, 0.51) | | | 0.86 |  | 0.23 (-0.17, 0.63) | | | 0.26 |
| Left parietal gray matter volume | -0.37 (-1.86, 1.12) | | | 0.63 |  | 1.19 (-0.08, 2.46) | | | 0.07 |
| Left parietal white matter volume (log) | 0.00 (-0.01, 0.00) | | | 0.17 |  | 0.00 (0.00, 0.01) | | | 0.73 |
| Left putamen volume | -0.01 (-0.06, 0.05) | | | 0.84 |  | -0.07 (-0.12, -0.02) | | | 0.01 |
| Left subthalamic nucleus volume | -0.25 (-1.53, 1.03) | | | 0.70 |  | -0.18 (-1.27, 0.91) | | | 0.75 |
| Left temporal gray matter volume | 0.43 (-0.51, 1.38) | | | 0.37 |  | 0.16 (-0.64, 0.97) | | | 0.69 |
| Left temporal white matter volume (log) | 0.00 (-0.01, 0.01) | | | 0.82 |  | 0.00 (-0.00, 0.01) | | | 0.28 |
| Left thalamus volume | -0.03 (-0.10, 0.04) | | | 0.37 |  | -0.03 (-0.08, 0.03) | | | 0.38 |
| Right volume |  |  |  |  |  |  |  |  |  |
| Right caudate volume (log) | 0.01 (-0.00, 0.01) | | | 0.26 |  | 0.00 (-0.01, 0.00) | | | 0.28 |
| Right cerebellum volume | -0.05 (-0.86, 0.77) | | | 0.91 |  | -0.18 (-0.87, 0.52) | | | 0.62 |
| Right fornix volume | -8.90 (-21.65, 3.85) | | | 0.17 |  | -3.95 (-14.84, 6.94) | | | 0.48 |
| Right frontal gray matter volume | 0.26 (-1.59, 2.11) | | | 0.78 |  | 0.71 (-0.87, 2.29) | | | 0.38 |
| Right frontal white matter volume | -1.16 (-2.31, 0.00) | | | 0.05 |  | -0.04 (-1.03, 0.95) | | | 0.93 |
| Right globus pallidus volume | -6.80 (-23.17, 9.58) | | | 0.42 |  | -12.05 (-26.00, 1.90) | | | 0.09 |
| Right hippocampus volume | 0.00 (-0.06, 0.06) | | | 0.99 |  | -0.01 (-0.06, 0.04) | | | 0.71 |
| Right lateral ventricle volume (log) | 0.05 (0.01, 0.08) | | | 0.01 |  | 0.02 (-0.01, 0.05) | | | 0.18 |
| Right occipital gray matter volume | 0.49 (-0.33, 1.32) | | | 0.24 |  | 0.45 (-0.25, 1.15) | | | 0.21 |
| Right occipital white matter volume (log) | 0.00 (-0.01, 0.01) | | | 0.60 |  | 0.00 (0.00, 0.01) | | | 0.45 |
| Right parietal gray matter volume | -0.60 (-1.90, 0.70) | | | 0.36 |  | 1.15 (0.04, 2.25) | | | 0.04 |
| Right parietal white matter volume (log) | 0.00 (-0.01, 0.00) | | | 0.16 |  | 0.00 (-0.00, 0.01) | | | 0.44 |
| Right putamen volume | 0.01 (-0.05, 0.07) | | | 0.72 |  | -0.06 (-0.11, -0.01) | | | 0.02 |
| Right subthalamic nucleus volume | 0.33 (-0.95, 1.62) | | | 0.61 |  | -0.51 (-1.61, 0.59) | | | 0.36 |
| Right temporal gray matter volume | 0.01 (-0.93, 0.96) | | | 0.98 |  | -0.23 (-1.03, 0.58) | | | 0.58 |
| Right temporal white matter volume (log) | 0.00 (-0.01, 0.00) | | | 0.32 |  | 0.00 (0.00, 0.01) | | | 0.27 |
| Right thalamus volume | -0.02 (-0.09, 0.04) | | | 0.52 |  | -0.02 (-0.07, 0.04) | | | 0.53 |

* CES-D-L: Loneliness scale from Center for Epidemiologic Studies Depression Scale; MRI: Magnetic Resonance Imaging; UCLA-LS: UCLA-Loneliness Scale

## Supplementary Table 7. Depressive symptom adjusted model

| MRI metrics | UCLA-LS | | CES-D-L | |
| --- | --- | --- | --- | --- |
|  | β (95% CI) | p-value | β (95% CI) | p-value |
| K-MMSE-2 | 1.471 (0.655 - 3.303) | 0.350 | 1.321 (0.541 - 3.225) | 0.541 |
| SNSB-C |  |  |  |  |
| Attention | 0.993 (0.598 - 1.649) | 0.980 | 0.715 (0.431 - 1.186) | 0.194 |
| Language | 1.351 (0.662 - 2.757) | 0.409 | 1.615 (0.802 - 3.252) | 0.180 |
| Visuospatial function | 1.337 (0.843 - 2.122) | 0.217 | 0.992 (0.618 - 1.592) | 0.973 |
| Memory | 1.279 (0.752 - 2.175) | 0.364 | 1.050 (0.606 - 1.819) | 0.862 |
| Executive function | 1.705 (0.856 - 3.394) | 0.129 | 1.583 (0.768 - 3.264) | 0.213 |
| Total | 1.707 (0.993 - 2.936) | 0.053 | 0.753 (0.411 - 1.378) | 0.833 |
| Brain volume |  |  |  |  |
| Total brain volume | -2.348 (-8.434 – 3.737) | 0.450 | -0.264 (-6.167 – 5.638) | 0.930 |
| Cerebral white matter volume | -4.471 (-9.816 - 0.874) | 0.102 | 0.991 (-4.200 – 6.182) | 0.708 |
| Cerebral gray matter volume | 1.812 (-2.207 - 5.831) | 0.377 | -0.982 (-4.880 – 2.916) | 0.622 |
| Subcortical gray matter volume | 0.311 (-0.457 - 1.079) | 0.427 | -0.274 (-1.018 – 0.471) | 0.471 |
| WMH volume (log) | 0.034 (-0.033 - 0.100) | 0.319 | 0.000 (-0.065 – 0.064) | 0.991 |
| Whole brain cortical thickness | 0.004 (-0.012 - 0.019) | 0.660 | 0.003 (-0.012 – 0.019) | 0.665 |
| Left volume | | | | |
| Left frontal gray matter volume | -0.240 (-2.196 - 1.716) | 0.810 | 0.132 (-1.765 – 2.028) | 0.892 |
| Left frontal white matter volume | -1.266 (-2.443 - -0.090) | 0.035 | -0.119 (-1.263 – 1.024) | 0.838 |
| Left hippocampus volume | 0.034 (-0.022 - 0.091) | 0.235 | 0.053 (-0.002 – 0.109) | 0.058 |
| Left caudate volume (log) | 0.004 (-0.005 - 0.014) | 0.368 | -0.005 (-0.014 – 0.004) | 0.288 |
| Left globus pallidus volume | -15.813 (-33.727 - 2.101) | 0.084 | -18.353 (-35.706 - -0.099) | 0.039 |
| Left putamen volume | 0.005 (-0.056 – 0.066) | 0.877 | -0.074 (-0.133 - -0.015) | 0.015 |
| Right volume | | | | |
| Right frontal gray matter volume | 0.0013 (-1.922 – 1.948) | 0.989 | 0.446 (-1.430 – 2.321) | 0.642 |
| Right frontal white matter volume | -1.223 (-2.431 - -0.015) | 0.048 | 0.030 (-1.145 – 1.204) | 0.961 |
| Right hippocampus volume | 0.015 (-0.044 – 0.074) | 0.613 | 0.017 (-0.040 – 0.074) | 0.564 |
| Right caudate volume (log) | 0.006 (-0.004 – 0.016) | 0.253 | -0.007 (-0.016 – 0.003) | 0.176 |
| Right globus pallidus volume | -5.027 (-22.147 – 12.094) | 0.565 | -12.102 (-28.683 – 4.478) | 0.153 |
| Right putamen volume | 0.023 (-0.040 – 0.085) | 0.480 | -0.061 (-0.122 – 0.000) | 0.049 |

*CES-D-L: Lonelieness scale from Center for Epidemiologic Studies Depression Scale; K-MMSE-2: Korean version of Mini-Mental State Examination 2; MRI: Magnetic Resonance Imaging; SNSB-C: Seoul Neuropsychological Screening Battery; UCLA-LS: UCLA-Loneliness Scale
** Age, sex, education level, hypertension, diabetes, CVD, depressive symptom were adjusted in multivariate analyses.

# Supplementary Figures

## Supplementary Figure 1. Scatter plot between UCLA-LS and CES-D-L


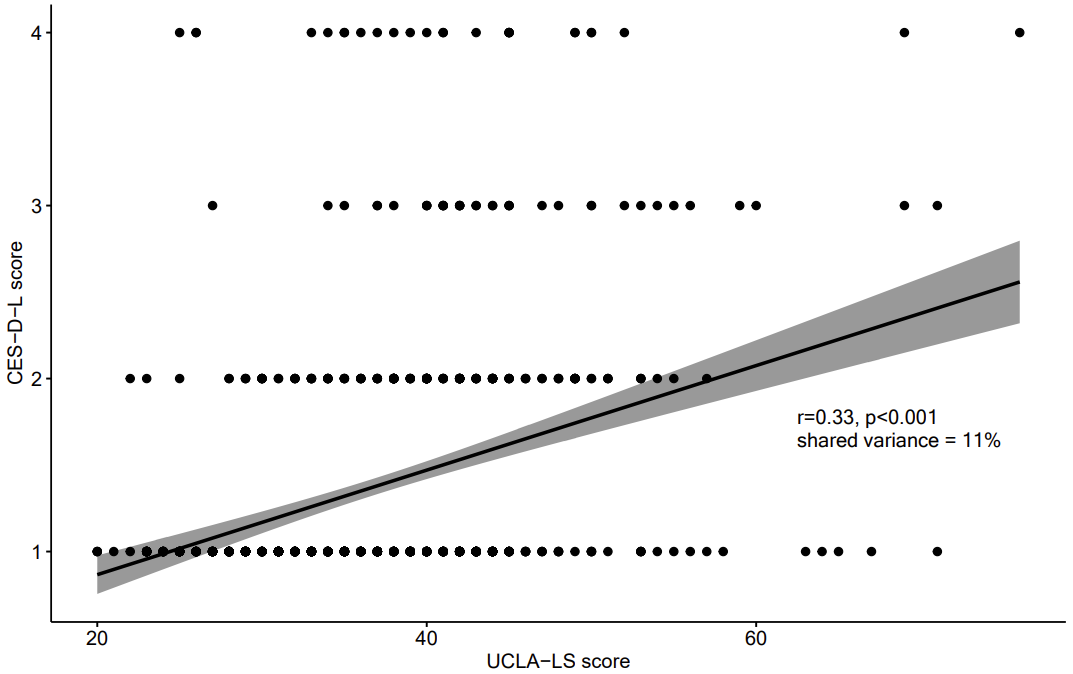


## Supplementary Figure 2. Scatter plots for UCLA-LS


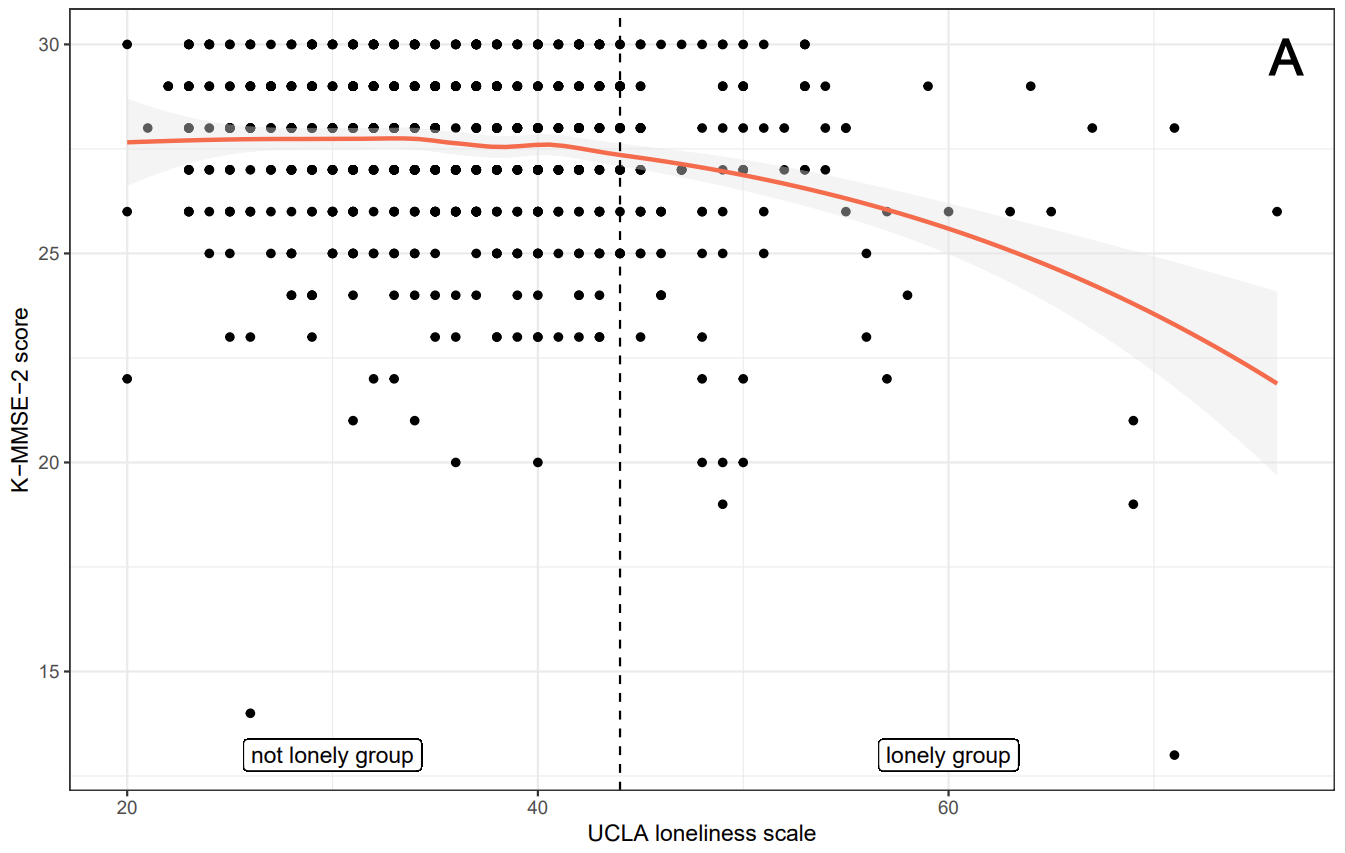


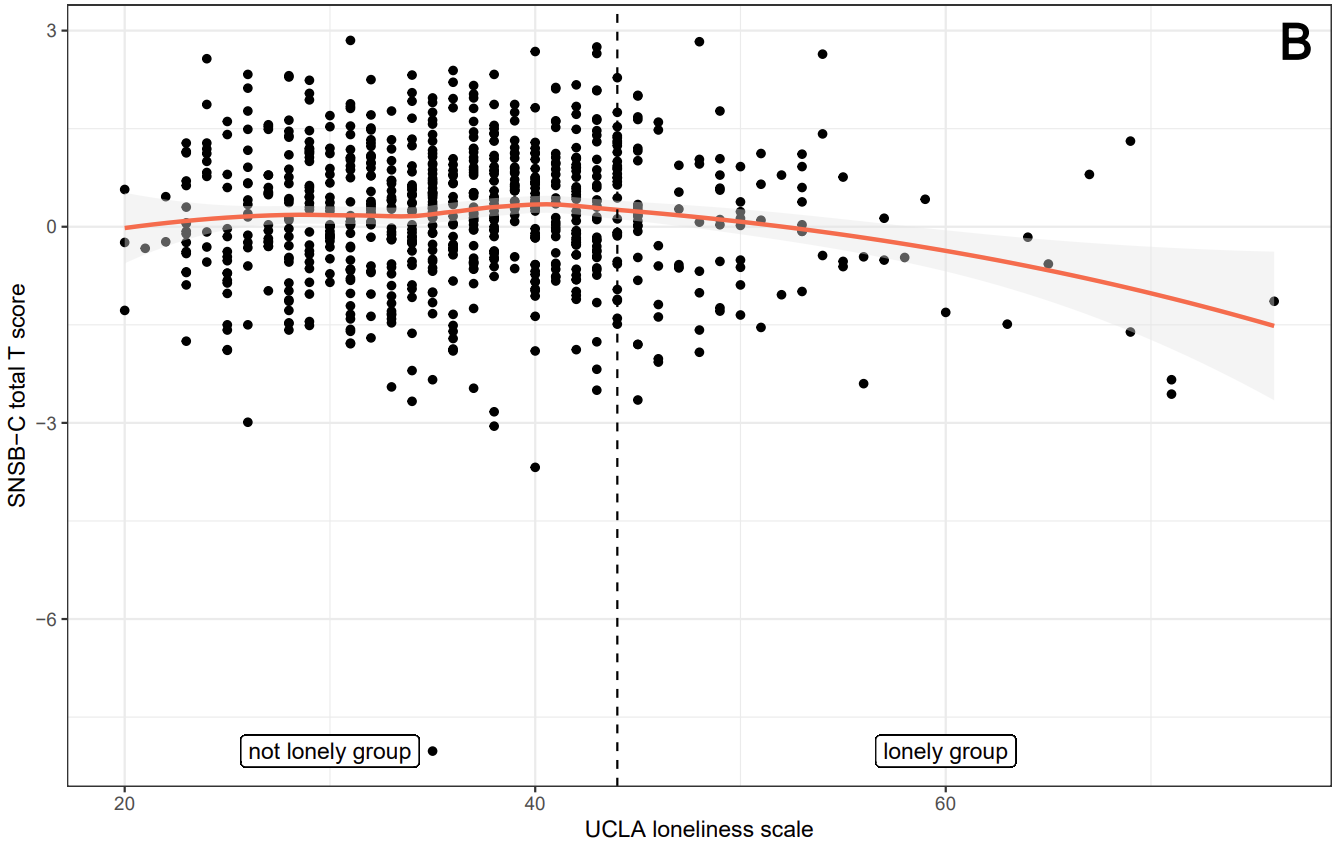


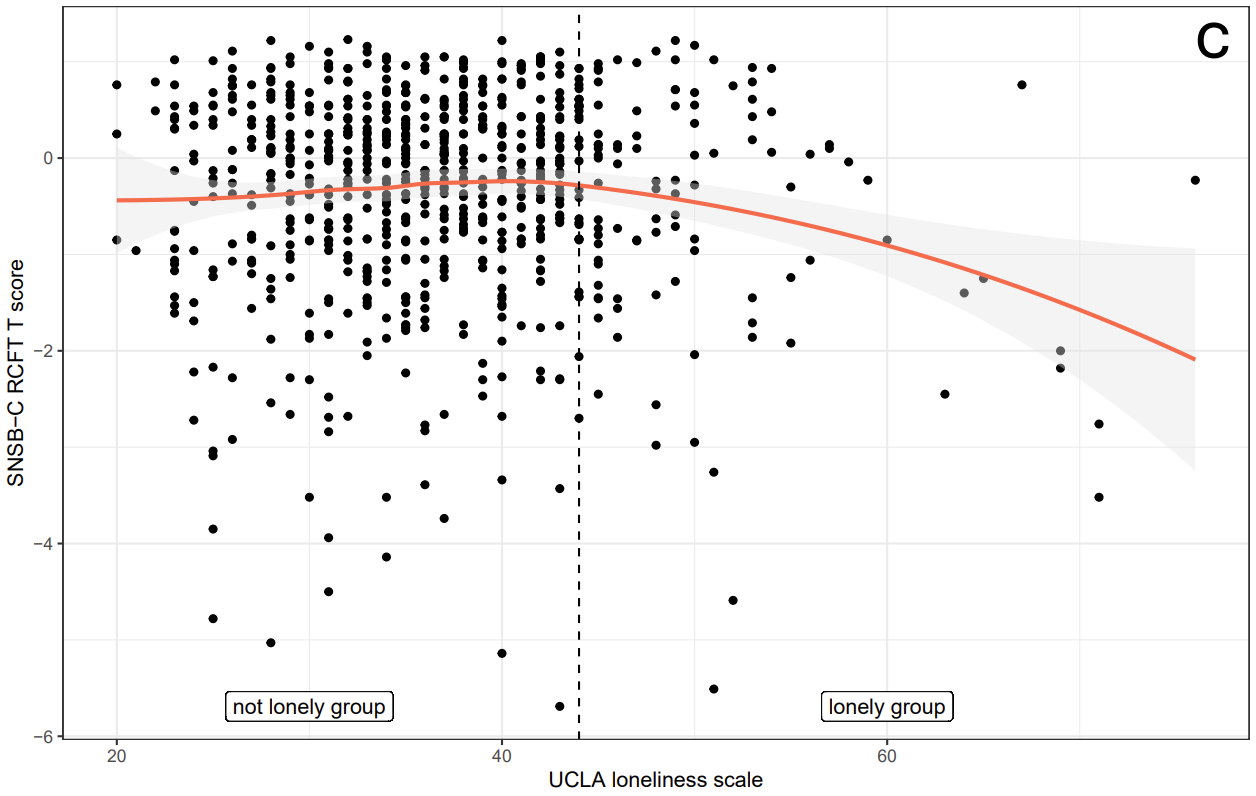


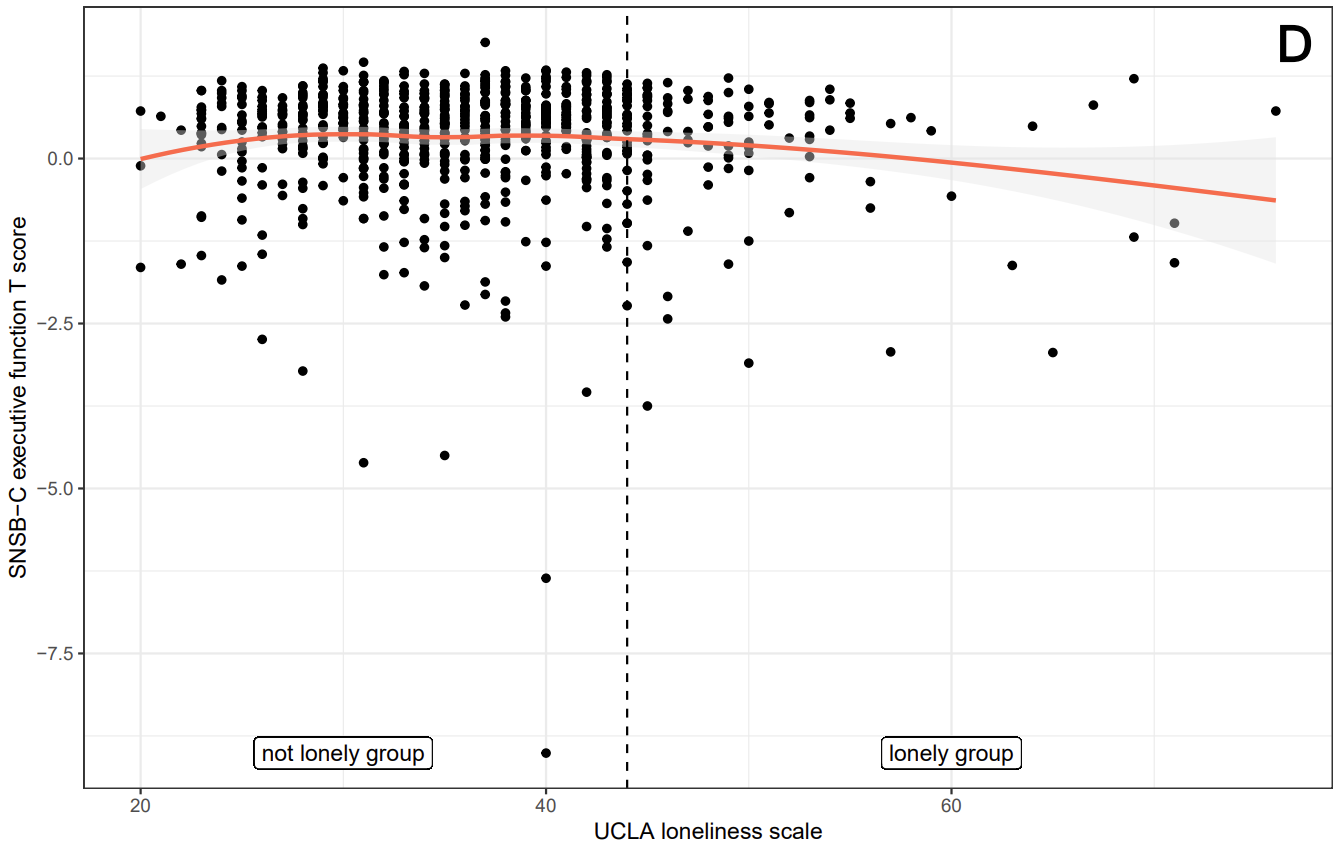


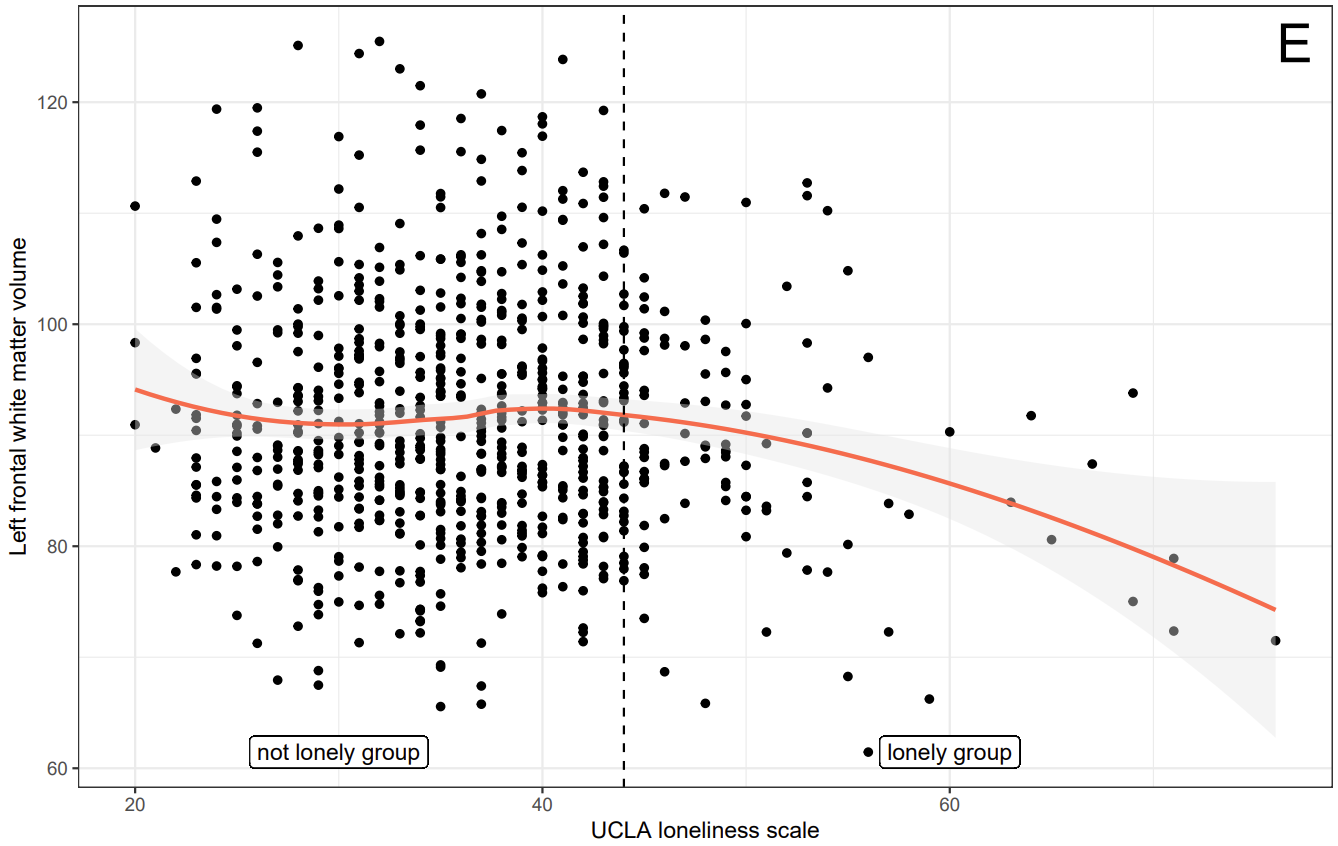


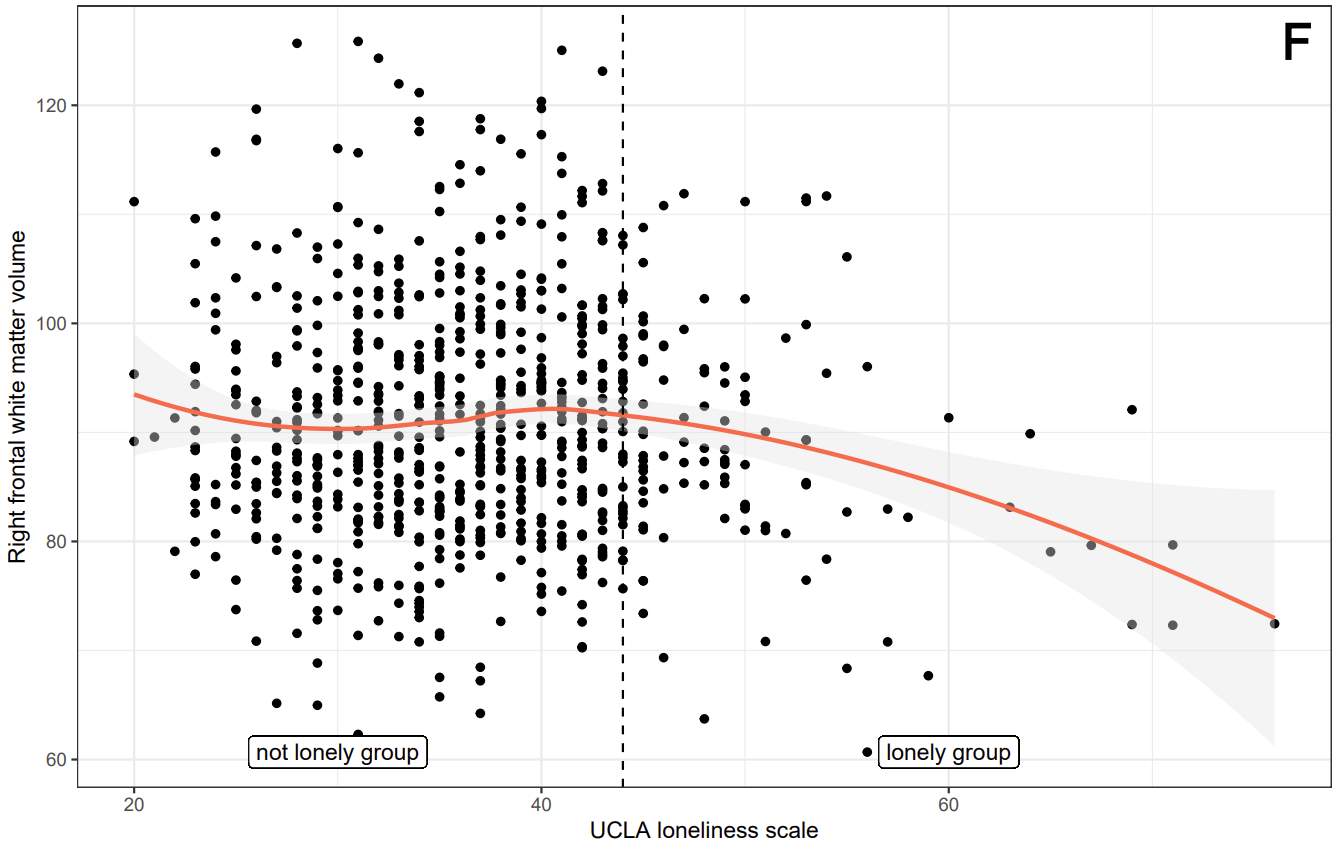


A: scatter plot for relationship between UCLA-LS and K-MMSE-2; B: scatter plot for relationship between UCLA-LS and SNSB-C total T score; C: scatter plot for relationship between UCLA-LS and visuospatial function; D: scatter plot for relationship between UCLA-LS and executive function; E: scatter plot for relationship between UCLA-LS and left frontal white matter volume; F: scatter plot for relationship between UCLA-LS and right frontal white matter volume

## Supplementary Figure 3. Violin plots for CES-D-L


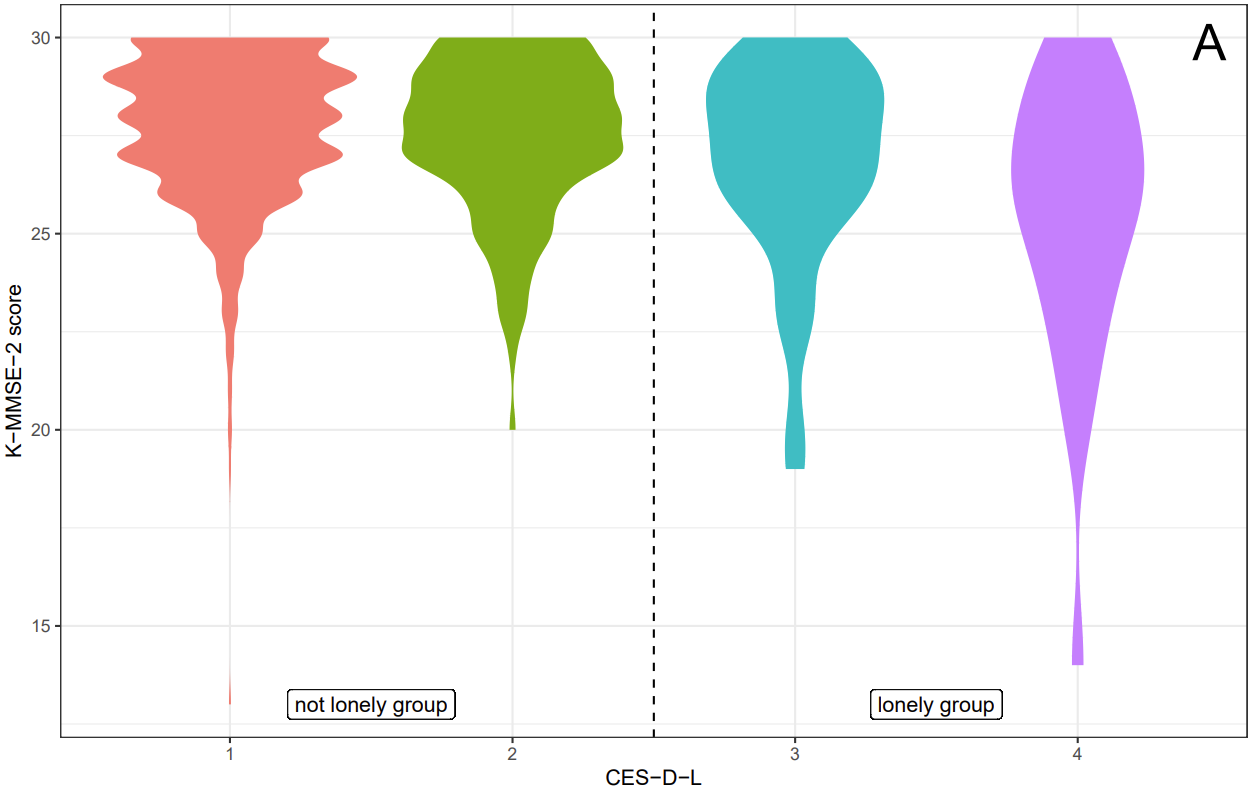


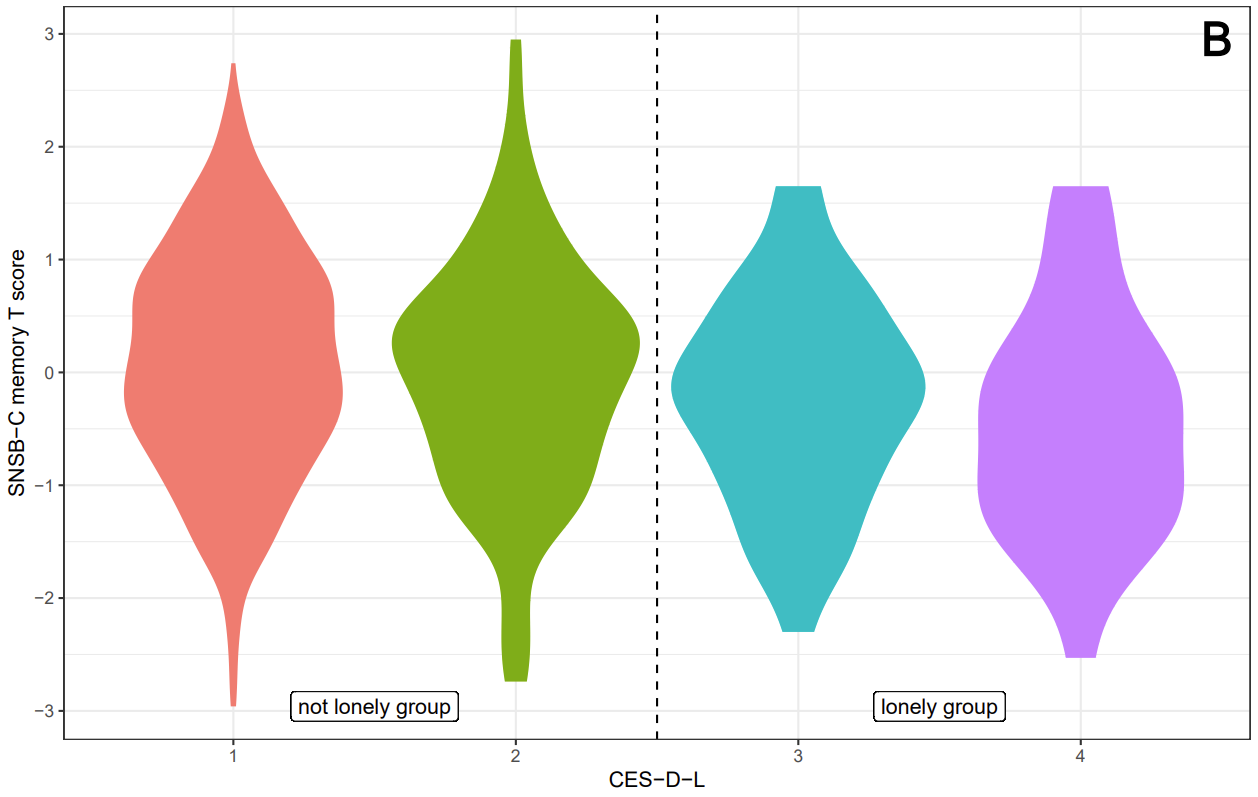


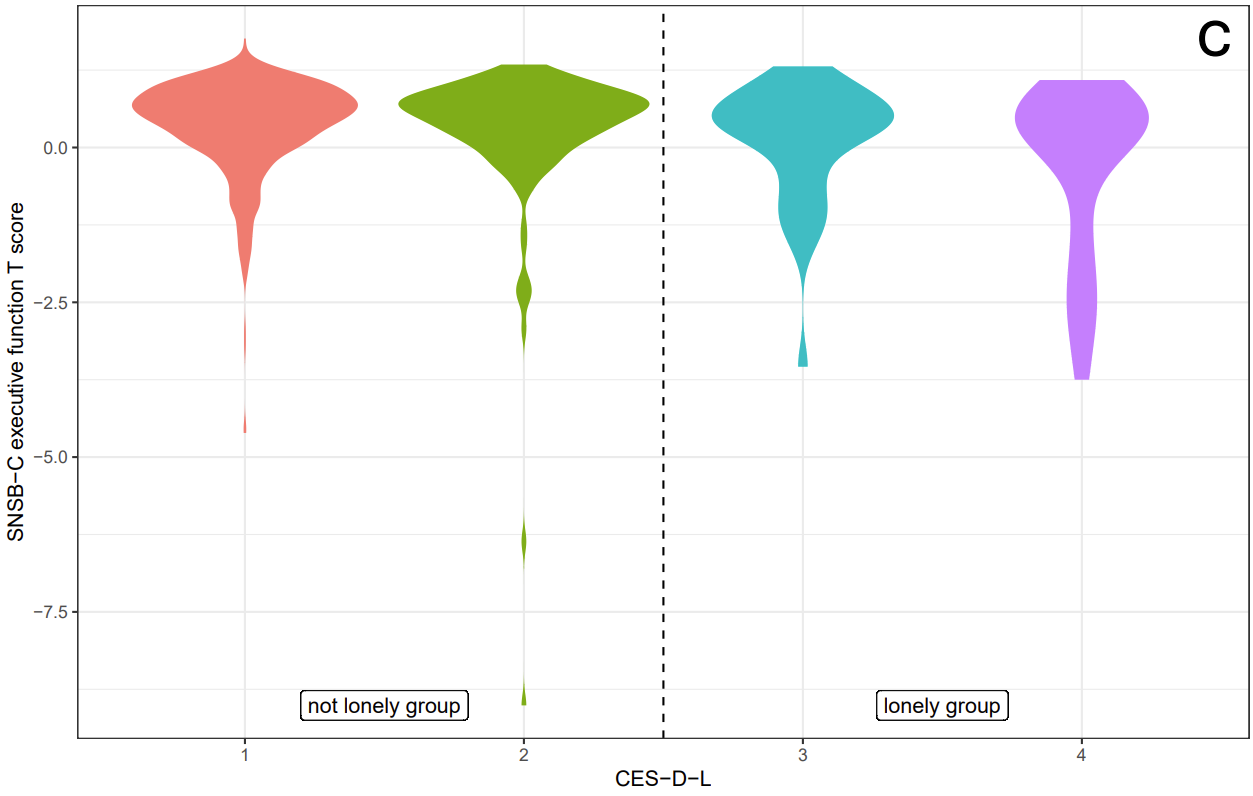


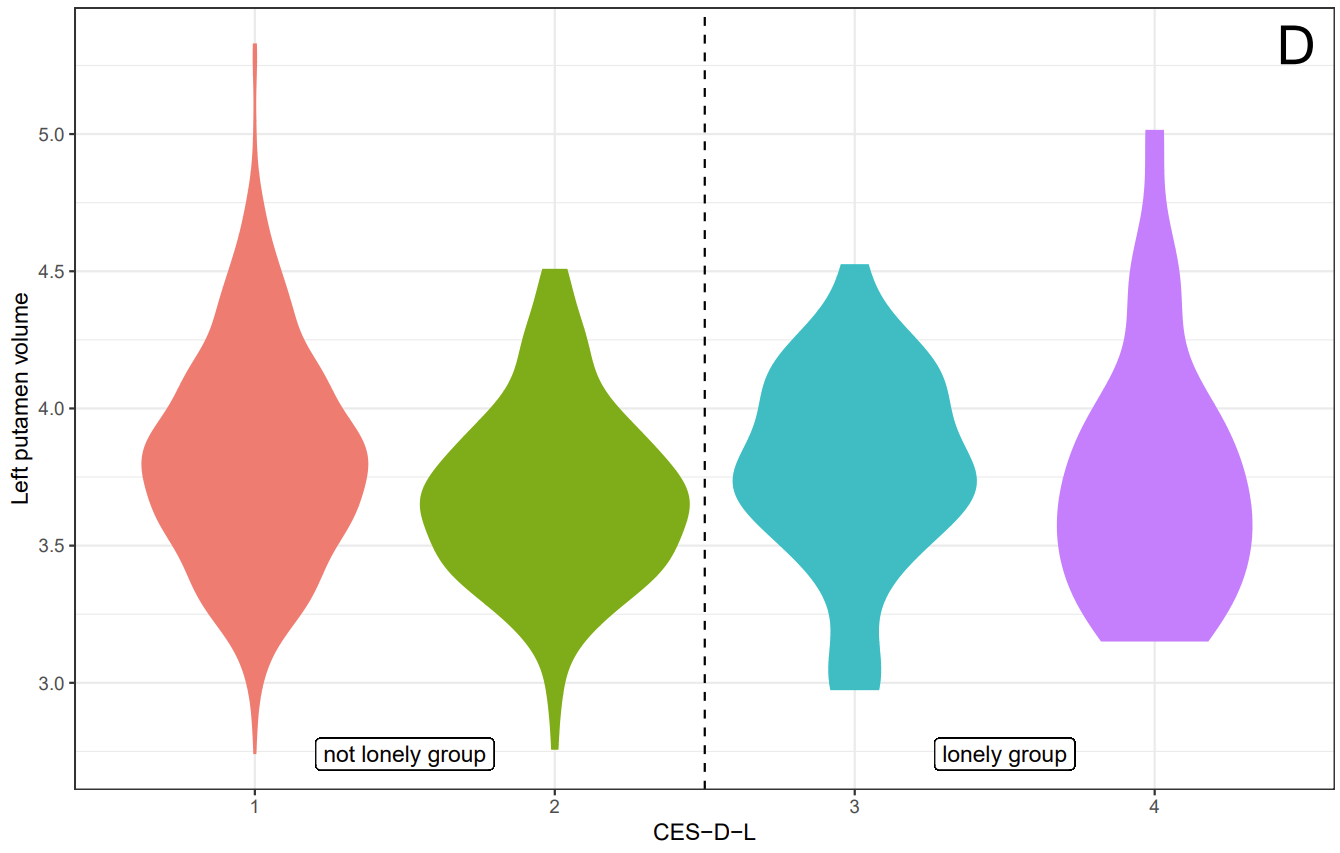


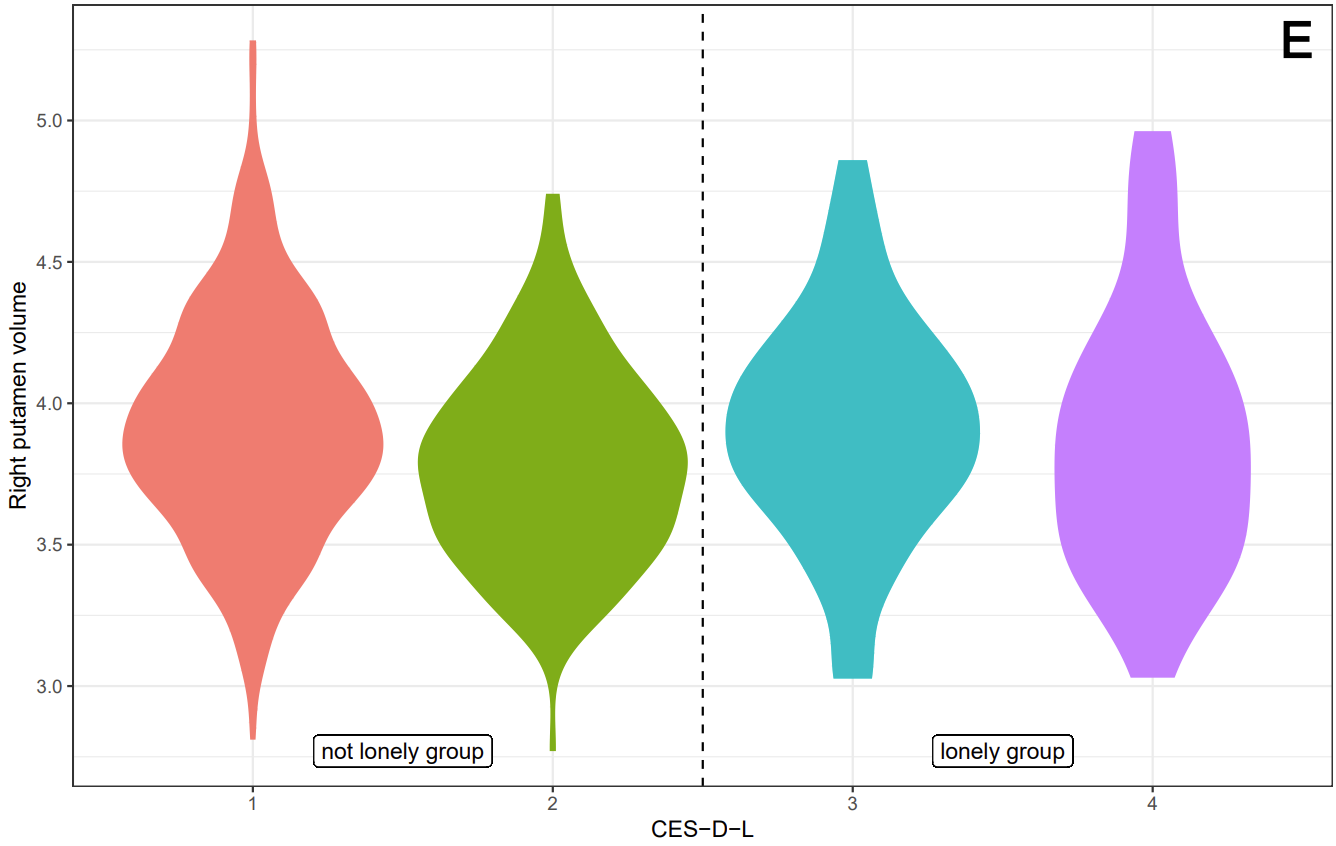


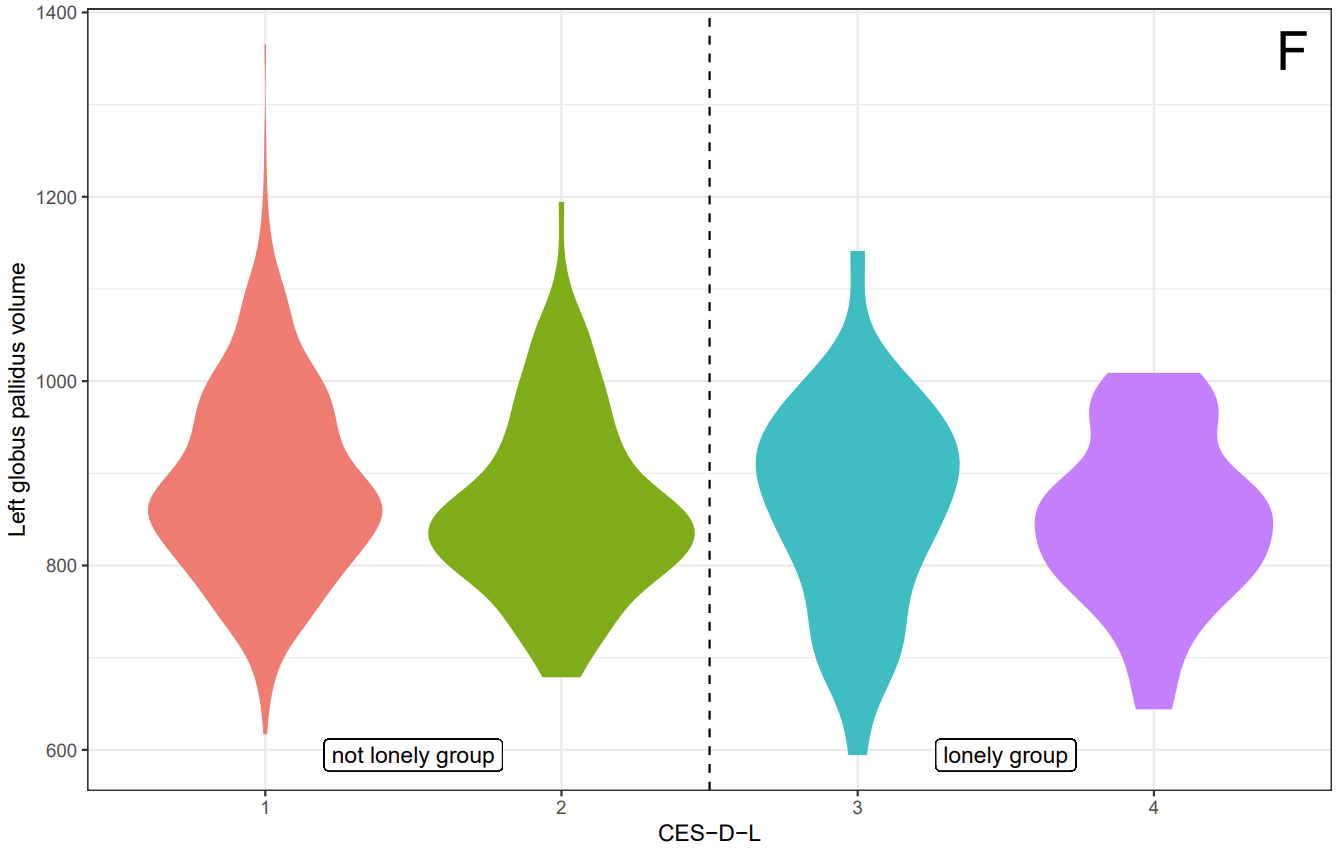


A: violin plot for relationship between CES-D-L and K-MMSE-2; B: violin plot for relationship between CES-D-L and memory function; C: violin plot for relationship between CES-D-L and executive function; D: violin plot for relationship between CES-D-L and left putamen volume; E: violin plot for relationship between CES-D-L and right putamen volume; F: violin plot for relationship between CES-D-L and left globus pallidus volume
